# Apoptotic vesicle-mediated senolytics requires mechanical loading

**DOI:** 10.7150/thno.98763

**Published:** 2024-08-06

**Authors:** Zhulin Xue, Yexiang Jiang, Bowen Meng, Lu Lu, Meng Hao, Yi Zhang, Songtao Shi, Zili Li, Xueli Mao

**Affiliations:** 1 Department of Oral and Maxillofacial Surgery, Peking University School and Hospital of Stomatology & National Center for Stomatology & National Clinical Research Center for Oral Diseases & National Engineering Research Center of Oral Biomaterials and Digital Medical Devices, Beijing 100081, China.; 2 Hospital of Stomatology, Guanghua School of Stomatology, Sun Yat-sen University, South China Center of Craniofacial Stem Cell Research, Guangdong Provincial Key Laboratory of Stomatology, Guangzhou 510055, China.; 3 Key Laboratory of Stem Cells and Tissue Engineering (Sun Yat-Sen University), Ministry of Education, Guangzhou 510080, China.

**Keywords:** apoptotic vesicles, apoptosis, mesenchymal stem cells, senescent cells, mechanical unloading

## Abstract

**Rationale:** Mechanical force plays crucial roles in extracellular vesicle biogenesis, release, composition and activity. However, it is unknown whether mechanical force regulates apoptotic vesicle (apoV) production.

**Methods:** The effects of mechanical unloading on extracellular vesicles of bone marrow were evaluated through morphology, size distribution, yield, and protein mass spectrometry analysis using hindlimb unloading (HU) mouse model. Apoptosis resistance and aging related phenotype were assessed using HU mouse model *in vivo* and cell microgravity model *in vitro.* The therapeutic effects of apoVs on HU mouse model were assessed by using microcomputed tomography, histochemical and immunohistochemical, as well as histomorphometry analyses. SiRNA and chemicals were used for gain and loss-of-function assay.

**Results:** In this study, we show that loss of mechanical force led to cellular apoptotic resistance and aging related phenotype, thus reducing the number of apoVs in the circulation due to down-regulated expression of Piezo1 and reduced calcium influx. And systemic infusion of apoVs was able to rescue Piezo1 expression and calcium influx, thereby, rescuing mechanical unloading-induced cellular apoptotic resistance, senescent cell accumulation.

**Conclusions:** This study identified a previously unknown role of mechanical force in maintaining apoptotic homeostasis and eliminating senescent cells. Systemic infusion of mesenchymal stem cell-derived apoVs can effectively rescue apoptotic resistance and eliminate senescent cells in mechanical unloading mice.

## Introduction

Mechanical force plays an important role in the maintenance of tissue health 1, 2. Loss of mechanical stimulation due to long term bed rest, immobilization, or spaceflight may cause cellular degeneration and various pathologies such as osteoporosis 3-5. Physical activity can only partially reduce mechanical unloading-induced bone loss 6. The detailed physiopathological mechanism of unloading-induced disorders remains poorly understood.

Mechanical unloading may be closely associated with aging due to vascular and metabolic changes, immune-neuroendocrine systemic alterations, muscle atrophy and bone loss 7-9. Senescent cells accumulate with aging and at pathogenic sites of various disorders, inducing the senescence-associated secretory phenotype (SASP) and ultimately leading to tissue dysfunction 10, 11. Developing interventions to clear these persistent senescent cells offers a promising strategy for treating multiple diseases and age-related conditions 12-14.

Extracellular vesicles, including apoptotic vesicles (apoVs), microvesicles, and exosomes, play a crucial role in communication and signal transduction between tissues 15, 16. Apoptosis is a universally conserved and critical physiological process of programmed cell death, essential for eliminating redundant, dysfunctional, or senescent cells. Billions of cells undergo apoptosis every day in the human body, and this process is increasingly understood as a vital metabolic activity that maintain tissue and organ homeostasis along with the generation of a substantial quantity of apoVs 17-19. ApoVs, which comprise a heterogeneous population of nanosized vesicles containing proteins, DNA, RNA, lipids, and metabolites, contribute to a variety of physiological and pathophysiological events 20-22. The release, composition, and activity of extracellular vesicles are affected by mechanical force 23. However, whether mechanical force regulates apoV production is largely unknown. In this study, we showed that mechanical unloading caused cellular apoptotic resistance, thus reducing the number of apoVs in the circulation due to down-regulated expression of Piezo1 and reduced calcium influx, ultimately leading to the accumulation of senescent cells in the bone marrow. Systemic infusion of apoVs was capable of rescuing disuse-induced osteoporosis *via* recovering apoptotic resistance and eliminating senescent cells.

## Results

### Mechanical unloading causes a reduction of apoVs in the bone marrow

**Figure 1:**
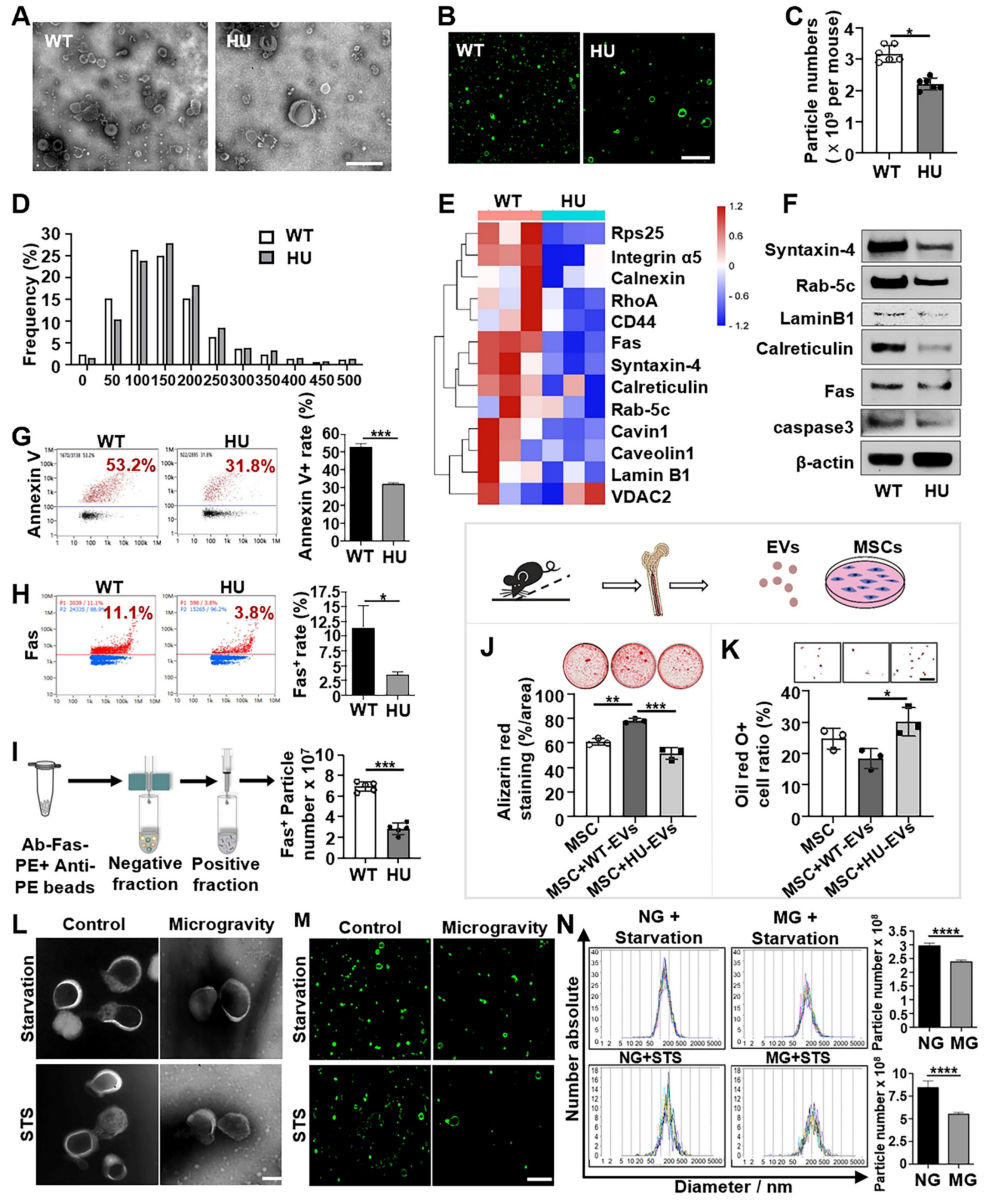
** Mechanical unloading reduces the number of apoVs in the bone marrow.** (**A**) Representative transmission electron microscope (TEM) image showing the morphology of bone marrow EVs from freely moving wildtype and hindlimb unloading mice. Bone marrow EVs were isolated from the femurs of freely moving wildtype or 2-week hindlimb unloading mice using sequential centrifugation. Scale bar, 500 nm. (**B**) Elyra 7 Lattice SIM images of PKH26-labeled bone marrow EVs from freely moving wildtype or hindlimb unloading mice. Scale bar, 5 µm. (**C**) Hindlimb unloading significantly reduced the particle numbers of bone marrow EVs. Particle numbers of bone marrow EVs were measured using nanoparticle tracking analysis. *n* = 5. (**D**) Nanoparticle tracking analysis showing the size distributions of bone marrow EVs from freely moving wildtype and hindlimb unloading mice. (**E**) Heatmaps showing that hindlimb unloading downregulated the apoV-specific biomarkers expression in bone marrow EVs. Each row represents one gene, and each column represents one of 3 samples. (**F**) Western blot analysis confirmed that hindlimb unloading downregulated the expression of apoV-specific markers Syntaxin-4, Rab-5C, Lamin B1, calreticulin, Fas, and caspase3 in bone marrow EVs. β-Actin was used as a protein loading control. (**G**) Nano flow cytometry showed that hindlimb unloading significantly reduced Annexin V-positive EVs in the bone marrow. Right panel shows quantification of Annexin V positive EVs, *n* = 5. (**H**) Nano flow cytometry showed that hindlimb unloading significantly reduced Fas-positive EVs in the bone marrow. Right panel shows quantification of Fas positive EVs, *n* = 5. (**I**) Nanoparticle tracking analysis the enrichment of Fas-positive EVs *via* magnetic bead sorting. (**J**) Compared to freely moving wildtype bone marrow EVs, hindlimb unloading EVs showed a reduced capacity to induce MSCs to form mineralized nodules under osteogenic conditions, assessed by alizarin red staining, *n* = 3. (**K**) Compared to freely moving wildtype bone marrow EVs, hindlimb unloading EVs showed an increased capacity to induce MSCs to differentiate into adipocytes under adipogenic conditions, as assessed by Oil red O staining, *n* = 3. Scale bar, 100 μm. (**L**) Representative TEM image showing the morphology of MSC-derived apoVs under normal gravity or microgravity after starvation in serum-free medium for 48 hours (upper) or 100 nM STS treatment for 24 hours (lower). A rotary cell culture system was used to simulate microgravity. ApoVs were isolated from the medium of apoptotic MSCs using sequential centrifugation. Scale bar, 100 nm. (**M**) Elyra 7 Lattice SIM images of PKH26-labeled MSC-apoVs induced by starvation and STS under normal gravity or microgravity. Scale bar, 5 µm. (**N**) Nanoparticle tracking analysis showed that microgravity significantly reduced the particle numbers of MSC-apoVs. *n* = 5. All data are shown as means ± SD. *P < 0.05, **P < 0.01, ***P < 0.001, and ****P < 0.0001.

Hindlimb unloading (HU) in mice is a well-established experimental model for simulating the physiological effects of spaceflight and microgravity environments. EVs have emerged as important regulators of intercellular and interorgan communication, so we investigated the changes in bone marrow EVs under mechanical unloading conditions. We isolated the bone marrow EVs from wild-type freely moving mice (WT-EVs) and 2-week post-HU mice (HU-EVs) as previously described 24, 25. Examination of isolated EVs by transmission electron microscopy (TEM) and super-resolution structured illumination microscopy (SIM) showed particles with diverse sizes and morphologies, including the expected “cup-shaped” morphology in both preparations (Figure 1A, B). Additionally, western blot analysis showed that isolated EVs expressed common EV markers, including TSG101, CD9, and CD81 (Figure S1A). Nanoparticle tracking analysis (NTA) revealed a significant reduction in total bone marrow EV numbers and a larger mean particle size in the HU-EV group when compared to the WT-EV group (Figure 1C, D and Figure S1B).

To further assess whether mechanical unloading affects the cargo and components of bone marrow EVs, the proteomic profiles of HU-EVs and WT-EVs were compared using Q-Exactive HF X to acquire mass spectrometry (MS) data in data-independent acquisition (DIA) mode. The details of the identified proteins are listed in Figure S2. ApoVs play pivotal roles in maintaining organ and tissue homeostasis. Notably, among the 13 apoV-specific markers identified previously 26, 12 were downregulated in HU-EVs (Figure 1E). The primary markers of apoVs, including transmembrane proteins (Syntaxin-4), cytosolic proteins (Rab-5C), intracellular compartment-associated proteins (Lamin B1, calreticulin) and apoptotic markers (Fas, caspase3) were consistently downregulated in HU-EVs, as verified by Western blot analysis (Figure 1F). We used nanoflow cytometric analysis to confirm that HU-EVs exhibited reduced levels of phosphatidylserine (PtdSer, shown by Annexin V binding) and Fas than WT-EVs (Figure 1G, H). Additionally, magnetic beads separation of Fas-positive EVs showed a significantly decrease in the total number of Fas-positive particles in the HU-EV group, as assessed by NTA (Figure 1I). Next, we examined the function of HU-EVs by treating bone marrow MSCs, which were characterized by flow cytometry (Figure S1C). We showed that HU-EV treatment significantly reduced mineralized nodule formation and enhanced adipocyte formation in MSCs when compared to the WT-EV group (Figure 1J, K). These findings indicate that HU leads to a decrease in both the number and quality of apoVs in the bone marrow.

To further assess whether mechanical unloading affects the production of apoVs *in vitro*, we used the NASA-engineered rotary cell culture system (RCCS) to simulate microgravity (MG). By dynamically altering the orientation of cells relative to gravity, clinostats effectively reduced the gravitational field to near zero over each revolution, thereby minimizing the influence of gravitational sedimentation. We cultured MSCs under MG and normal gravity (NG) conditions with low levels staurosporine (STS, 0.1 µM) for 24 h or under starvation conditions in serum-free medium for 48 h to induce apoptosis. ApoVs were isolated from apoptotic MSCs using the optimized gradient centrifugation protocol 27 and characterized in terms of morphology, concentration and size distribution (Figure 1L-M). Our results showed that the number of apoVs derived from MG-MSCs was significantly lower than from NG-MSCs (Figure 1N). These data suggest that mechanical force is required for maintaining apoV production *in vitro*.

### Mechanical unloading induces apoptotic resistance in bone marrow cells

**Figure 2:**
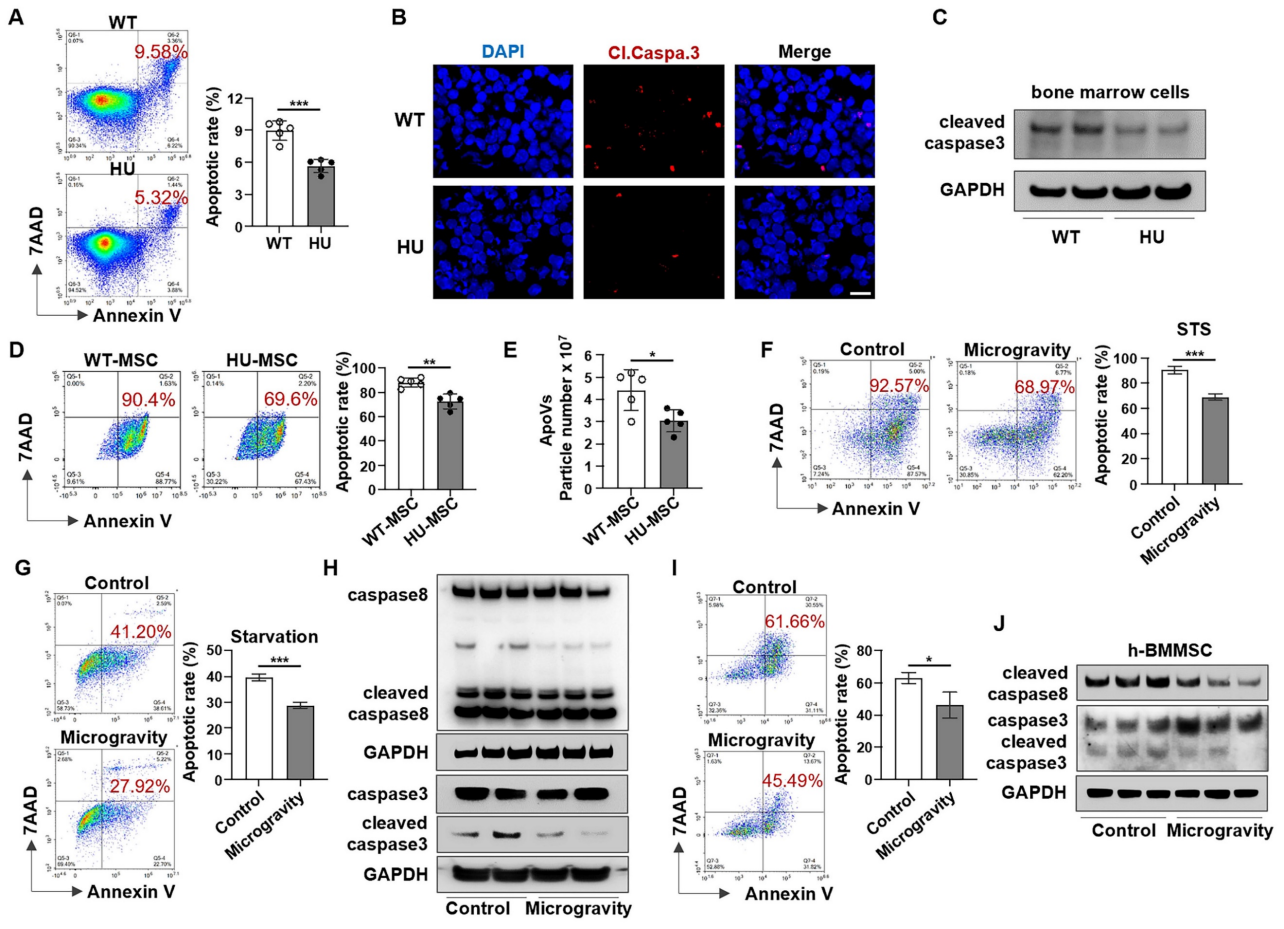
** Mechanical unloading induces bone marrow cell resistance to apoptosis.** (**A**) Flow cytometric analysis showed that the apoptotic rate of total bone marrow cells was significantly decreased in hindlimb unloading mice compared to freely moving wildtype mice, *n* = 5. (**B**) Immunofluorescence staining of distal femurs showed that cleaved caspase-3 expression was significantly decreased in hindlimb unloading mice compared to freely moving wildtype mice. Scale bar, 10 μm. (**C**) Western blot analysis showed that bone marrow cells from hindlimb unloading mice expressed a lower level of cleaved-caspase3 than those from freely moving wildtype mice. Bone marrow cells were isolated from femurs. GAPDH was used as a protein loading control. (**D**) Flow cytometric analysis showed that MSCs derived from hindlimb unloading mice had lower STS-induced apoptotic rate when compared to freely moving wildtype mice, *n* = 5. (**E**) Nanoparticle tracking analysis showed that hindlimb unloading reduced the particle numbers of MSC-apoVs after induced by STS, *n* = 5. (**F**) Flow cytometric analysis showed that the apoptotic rate of MSCs induced by STS (100 nM for 24h) was significantly decreased under microgravity compared to normal gravity, *n* = 3. (**G**) Flow cytometric analysis showed that the apoptotic rate of MSCs induced by starvation was significantly decreased under microgravity compared to normal gravity, *n* = 3. (**H**) Western blot showed that the levels of cleaved-caspase3 and cleaved-caspase8 in starvation-induced MSCs were decreased under microgravity. GAPDH was used as a protein loading control. (**I**) Flow cytometric analysis showed that the apoptotic rate of human bone marrow mesenchymal stem cells (hBMMSCs) induced by starvation was significantly decreased under microgravity compared to normal gravity, *n* = 3. (**J**) Western blot showed that the levels of cleaved-caspase 3 and cleaved-caspase 8 decreased under microgravity in starvation-induced hBMMSCs. GAPDH was used as a protein loading control. All results are representative of data generated in at least three independent experiments. All data are shown as means ± SD. *P < 0.05, **P < 0.01, ***P < 0.001, and ****P < 0.0001.

The reduction in apoVs in the bone marrow further led us to hypothesize that mechanical unloading affects the apoptosis of bone marrow cells. We isolated bone marrow cells from the femurs of HU mice. Interestingly, flow cytometric analysis revealed that the apoptotic rate of bone marrow cells in HU mice was significantly lower compared to that in WT mice (Figure 2A). Furthermore, we found that HU repressed apoptosis in subsets of bone marrow cells and decreased the number of Annexin V-positive apoptotic cells in the bone marrow in a time-dependent manner (Figure S3). In addition, HU markedly downregulated cleaved caspase-3 expression in bone marrow cells, as assessed by immunofluorescence staining and Western blotting (Figure 2B, C). To further investigate whether mechanical unloading affects the response to apoptotic induction in bone marrow cells, we isolated MSCs from HU mice and assessed their reactions to apoptotic stimuli. MSCs derived from HU mice showed a significantly lower apoptotic rate (Figure 2D) and reduced production of apoVs (Figure 2E) after STS induction.

To confirm our *in vivo* findings and further define the role of mechanical unloading in MSC apoptosis, MSCs were cultured under NG or MG and simultaneously treated with 100 nM STS for 48 h to induce apoptosis. The rates of MSC apoptosis in the MG groups were significantly lower than those in the NG groups (Figure 2F). Results consistent with these were obtained when the apoptotic process was alternatively induced by starvation (Figure 2G). Western blot analysis showed lower protein levels of cleaved caspase-3 and cleaved caspase-8 in MG-MSCs (Figure 2H). In addition, we found that MG also repressed apoptosis and downregulated cleaved caspase-3 and cleaved caspase-8 expression in human bone marrow mesenchymal stem cells (hBMMSCs) (Figure 2I, J). Together, these data suggest that mechanical unloading induces apoptotic resistance in bone marrow cells.

### Mechanical force maintains apoptotic metabolism of MSCs *via* Piezo1-mediated calcium influx

**Figure 3:**
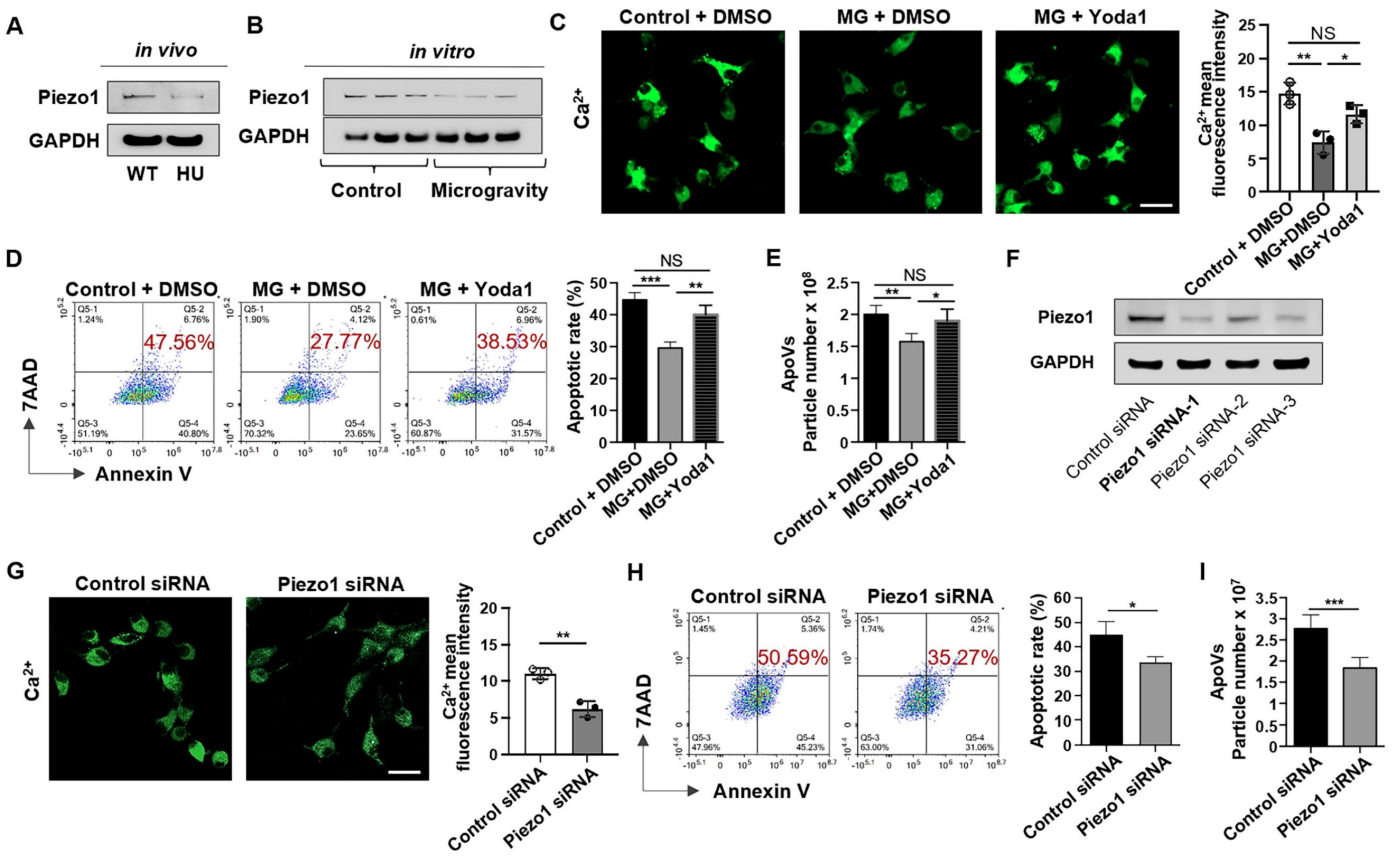
** Mechanical unloading reduces MSC apoptosis *via* inhibiting Piezo1 expression and calcium influx.** (**A**) Western blot analysis showed that mechanical unloading downregulated Piezo1 expression in MSCs. (**B**) Western blot analysis showed that MSCs under microgravity expressed a lower level of Piezo1 than those under normal gravity. The rotary cell culture system was used to simulate microgravity for 48 hours. GAPDH was used as a protein loading control. (**C**) Representative fluorescence images of intracellular Ca^2+^ showed that Yoda1 treatment (a Piezo1 chemical activator, 10 nm/ml) significantly rescued the decrease in intracellular Ca^2+^ under microgravity. Cytosolic Ca^2+^ was measured using Fluo-8AM. Scale bar, 20 μm. (**D**) Flow cytometric analysis showed that Yoda1 treatment rescued the decreased MSC apoptosis induced by starvation under microgravity, *n* = 3. (**E**) Nanoparticle tracking analysis showed that Yoda1 treatment rescued the reduced particle numbers of MSC-apoVs under microgravity. *n* = 3. (**F**) Western blot analysis showed that Piezo1 siRNA knockdown effectively inhibited Piezo1 expression in MSCs. (**G**) Piezo1 siRNA treatment blocked Ca^2+^ influx during starvation-induced MSC apoptosis. Representative fluorescence images of intracellular Ca^2+^ in MSCs and Piezo1 siRNA knockdown MSCs after starvation in serum-free medium for 48 hours. Cytosolic Ca^2+^ was measured using Fluo-8AM. *n* = 3. Scale bar, 20 μm. (**H**) Piezo1 siRNA treatment repressed starvation-induced MSC apoptosis. Representative flow cytometry plots and frequency of MSCs and Piezo1 siRNA knockdown MSCs after starvation in serum-free medium for 48 hours, *n* = 3. (**I**) Nanoparticle tracking analysis showed that Piezo1 siRNA treatment reduced the particle numbers of MSC-apoVs induced by starvation, *n* = 3. All data are shown as means ± SD. *P < 0.05, **P < 0.01, ***P < 0.001, and ****P < 0.0001. NS, not significant.

Piezo1 is a member of a novel class of mechanically activated cation channels (MACs) 28. It is a crucial factor in regulating mechanically mediated cellular behaviors, including cell apoptosis 29. To investigate whether Piezo1 plays a role in unloading-mediated resistance to apoptosis of bone marrow cells, we compared Piezo1 expression between MSCs sorted from WT and HU mice and found that Piezo1 was significantly down-regulated in HU mice (Figure 3A). To examine whether mechanical unloading directly influences Piezo1 expression in MSCs, we examined the expression level of Piezo1 in MSCs exposed to MG. Notably, we found that MG repressed the Piezo1 expression in cultured MSCs (Figure 3B). Because Piezo1 is a transmembrane ion channel that regulates mechanically induced Ca^2+^ influx 30, we hypothesized that the inhibition of Piezo1 under MG conditions decreased Ca^2+^ influx and repressed apoptosis. To test this hypothesis, we measured Ca^2+^ influx by assessing the fluorescence intensity of Ca^2+^. The results showed that MG decreased the intracellular Ca^2+^ concentration (Figure 3C). Notably, treatment with Yoda1, a previously identified Piezo1 chemical activator 31, rescued the decreased intracellular Ca^2+^ concentration induced by MG (Figure 3C), demonstrating that down-regulated Piezo1 reduced Ca^2+^ influx in MSCs when exposed to microgravity. Importantly, Yoda1 treatment rescued the decreased apoptotic rate of MSCs under MG (Figure 3D), as well as the reduced production of apoVs assessed by NTA (Figure 3E). In addition, knockdown of Piezo1 (Figure 3F) dramatically reduced Ca^2+^ influx during starvation-induced apoptosis (Figure 3G), mimicking the decreases in apoptotic MSCs and apoVs under MG (Figure 3H, I). Taken together, these data indicate that MG represses the apoptotic metabolism of MSCs *via* Piezo1-mediated calcium influx.

### Senescent cells accumulate in the bone marrow of unloading mice

**Figure 4:**
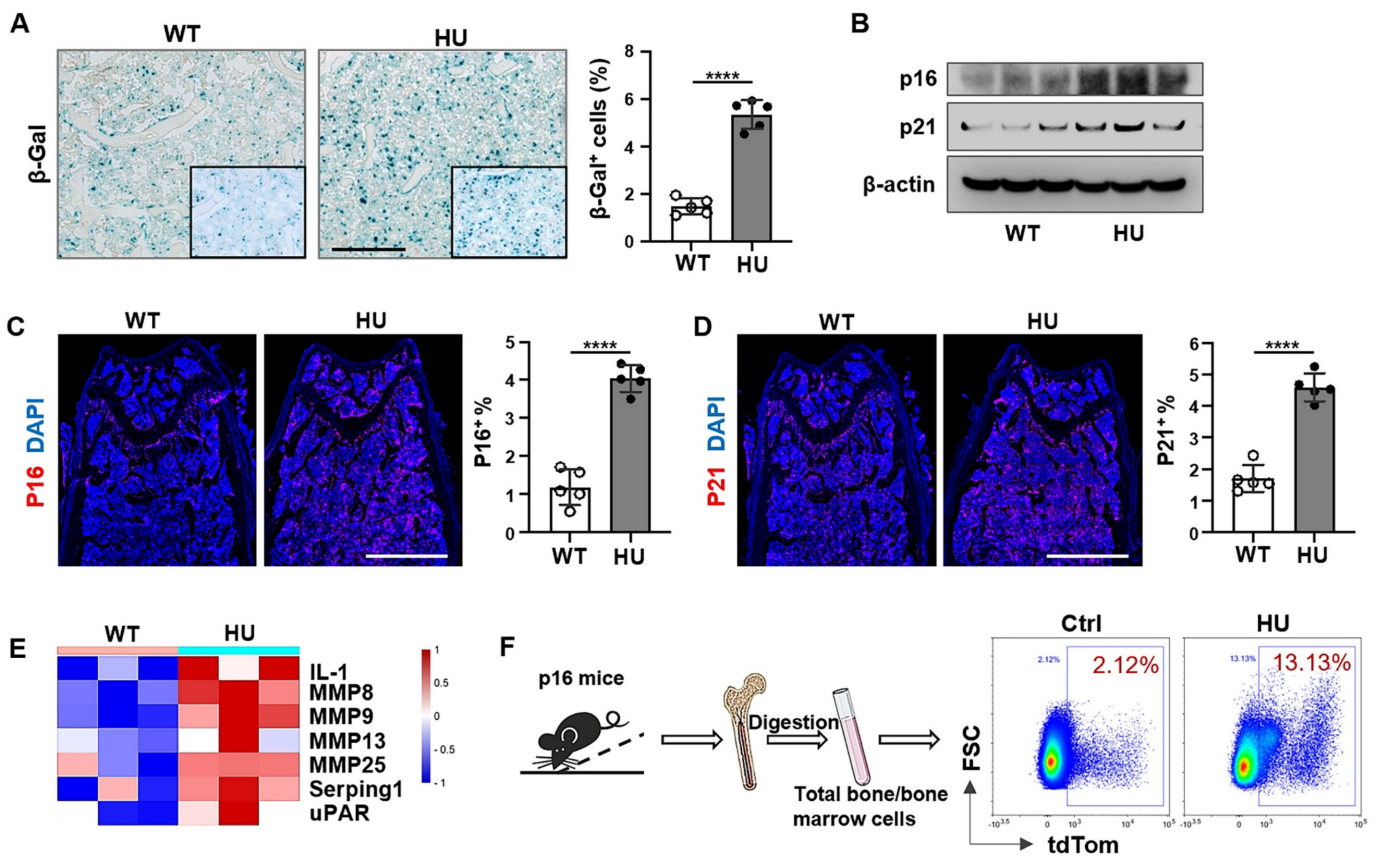
** Mechanical unloading leads to senescent cell accumulation in the bone marrow.** (**A**) SA-β-gal staining of senescent cells in distal femurs show that SA-β-gal^+^ cells significantly increased in hindlimb unloading mice compared to freely moving wildtype mice. Black dashed lines are higher magnification views of the boxed regions. Right panel shows quantification of SA-β-gal^+^ positive cells,* n* = 5. Scale bar, 200 μm. (**B**) Western blot analysis showed that hindlimb unloading upregulated p16 and p21 expression in bone marrow cells. β-Actin was used as a protein loading control. (**C** and** D**) Immunofluorescent staining showed that hindlimb unloading significantly increased p16^+^ and p21^+^ cells in distal femurs. Right panel showed quantification of p16 and p21 positive cells, respectively, *n* = 5. Scale bars, 1 mm. (**E**) Heatmaps showed that hindlimb unloading upregulated the SASP-related protein expression in bone marrow EVs. Each row represents one gene and each column represent one of 3 samples. (**F**) Schematic diagram illustrating the experimental procedure. Femoral bone and bone-marrow tissue were collected from p16-td^Tom^ mice and the isolated cells were subjected to flow cytometry analysis. All data are shown as means ± SD. *P < 0.05, **P < 0.01, ***P < 0.001, and ****P < 0.0001.

Apoptosis resistance is one of the most important characteristics of senescent cells, and the accumulation of senescent cells that resist apoptotic clearance in the body is the potential cause of age-related dysfunction. Next, we explored whether apoptosis resistance caused by mechanical unloading leads to the accumulation of senescent cells. Our results revealed that 2 weeks of HU induced an increase in the number of SA-β-Gal^+^ cells in the distal femur compared to freely moving WT mice (Figure 4A). Western blot analysis showed an increase in the expression of p16INK4a and p21, two biomarkers of cellular senescence, in HU mouse bone marrow cells (Figure 4B). Similarly, fluorescence analysis of femoral bone tissue sections showed that HU led to an increased number of p16^+^ and p21^+^ cells in the distal femur (Figure 4C, D). Senescent cells release a distinctive secretome composed of various bioactive molecules, collectively called SASP. These SASP factors are secreted both as single molecules and packaged within extracellular vesicles, contributing to the spread of senescent phenotype. Notably, several key SASP-related proteins, including IL-1, MMP family members, Serping1 and uPAR were upregulated in the HU-EVs compared to WT-EVs (Figure 4E), supporting the notion that HU induces senescent cell accumulation in the bone marrow. We also used a senescence reporter strain of mice containing p16^tdTom^, where p16INK4a-activated cells (tdTom^+^) were identifiable. Flow cytometry analysis revealed a gradual increase of tdTom^+^ senescent cells in the femoral bone in response to HU (Figure 4F). These data indicate that mechanical unloading induces an accumulation of senescent cells in the bone marrow.

### Clearance of senescent cells rescues osteoporosis in unloading mice

**Figure 5:**
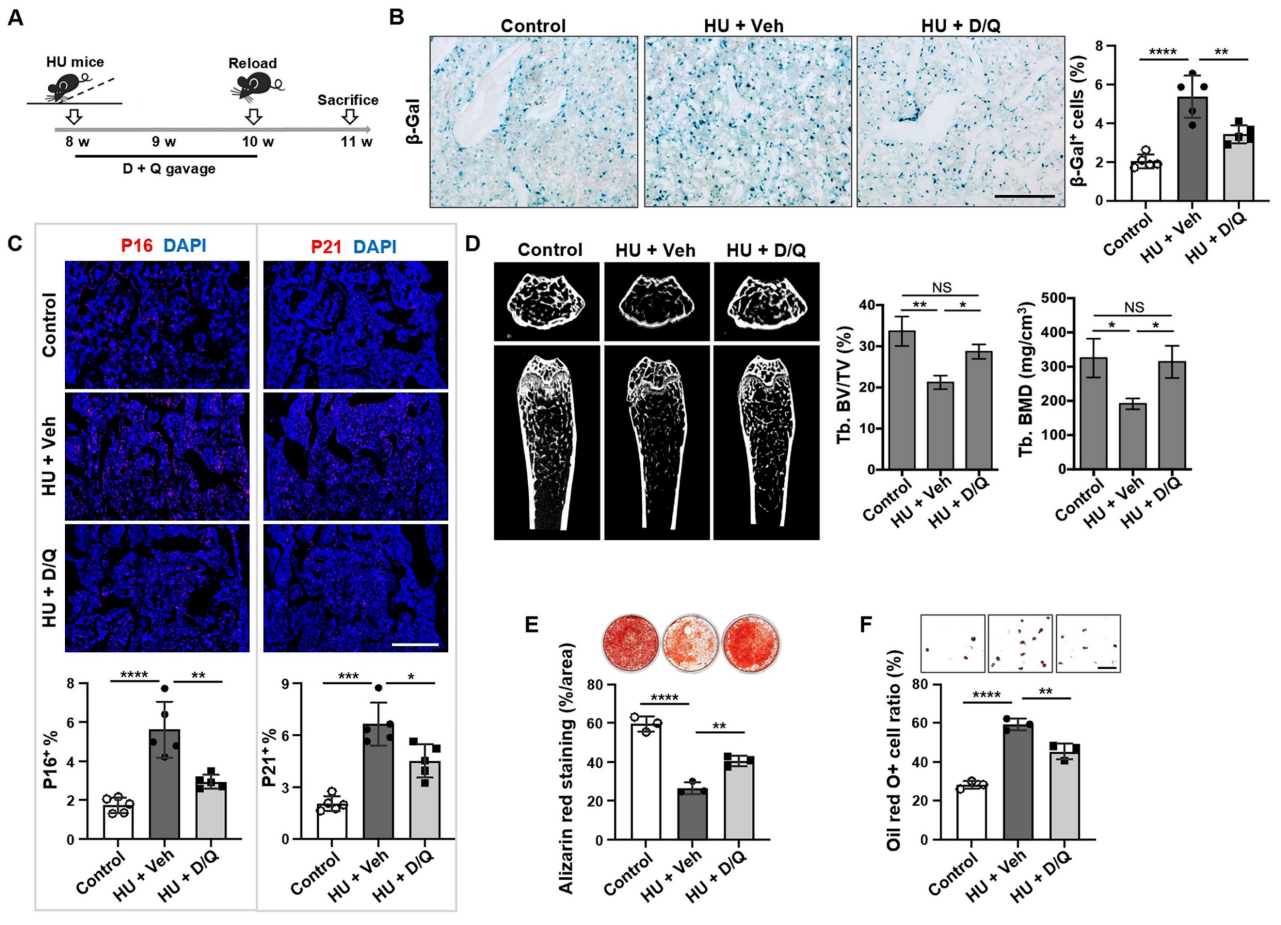
** Elimination of senescent cells by Senolytics rescues osteoporosis in unloading mice.** (**A**) Schematic diagram indicating the study design of D + Q (D, dasatinib, 5 mg/kg; and Q, quercetin, 50 mg/kg) treatment for hindlimb unloading mice: 8-week-old male C57BL/6 mice were randomized to receive either vehicle or D + Q treatment (once weekly by oral gavage) for 3 weeks,* n* = 5. (**B**) SA-β-gal staining of senescent cells in the distal femurs showed that D + Q treatment significantly reduced the SA-β-gal^+^ cells in hindlimb unloading mice when compared to the vehicle control group. Right panel shows quantification of SA-β-gal^+^ positive cells. Scale bar, 200 μm. (**C**) Immunofluorescence staining showed that D + Q treatment significantly reduced the p16^+^ and p21^+^ cells in the distal femurs of hindlimb unloading mice when compared to the vehicle control group. Lower panel showed quantification of p16 and p21 positive cells, respectively, *n* = 5. Scale bar, 500 μm. (**D**) After 3 weeks of D + Q treatment, the femurs of hindlimb unloading mice showed significantly increased bone mineral density (BMD) and bone volume/total volume (BV/TV), as assessed by microCT. (**E**) Compared MSCs from freely moving mice, hindlimb unloading MSCs showed reduced capacity to form mineralized nodules when cultured under osteogenic inductive conditions, assessed by alizarin red staining, *n* = 3. After 3 weeks of D + Q treatment, reduced mineralized nodule formation was rescued in hindlimb unloading MSCs, *n* = 3. (**F**) Compared to MSCs from freely moving mice, hindlimb unloading MSCs showed an increased capacity to differentiate into adipocytes when cultured under adipogenic inductive conditions, as assessed by Oil red O staining, *n* = 3. After 3 weeks of D + Q treatment, increased adipocyte formation was rescued in hindlimb unloading MSCs, *n* = 3. Scale bar, 100 μm. All data are shown as means ± SD. *P < 0.05, **P < 0.01, ***P < 0.001, and ****P < 0.0001. NS, not significant.

Senolytics, such as dasatinib (D; an FDA-approved tyrosine kinase inhibitor) and quercetin (Q; a flavanol present in many fruits and vegetables), cause senescent cells to undergo apoptosis *via* targeting senescent cell antiapoptotic pathways (SCAPs) 32. Therefore, we tested whether elimination of senescent cells could recover bone disorder in HU mice. The D + Q treatment was given at 0, 7, and 14 days post-HU, and the bones were analyzed at 21 days post-HU (Figure 5A). The clearance of senescent cells with D + Q was verified by reduced number of SA-β-gal-positive cells in D + Q-treated mouse bone tissue relative to vehicle-treated control group (Figure 5B). In addition, D + Q treatment significantly reduced the number of p16^+^, p21^+^, cleaved caspase 3^+^, and cleaved caspase 8^+^ cells in mouse bone tissue compared to vehicle-treated control group (Figure 5C, Figure S5). Importantly, D + Q administration to HU mice resulted in substantially better trabecular bone volume in the femur than in vehicle-treated control group (Figure 5D). Furthermore, D + Q treatment protected MSCs from HU-induced impairments *in vivo*, as evidenced by enhanced osteogenic differentiation and suppressed adipogenesis (Figure 5E, F). Collectively, these findings suggest that the impairments in MSCs and subsequent bone loss due to mechanical unloading are associated with the accumulation of senescent cells.

### Systemic apoV infusion eliminates senescent cells in mechanical unloading mice

**Figure 6:**
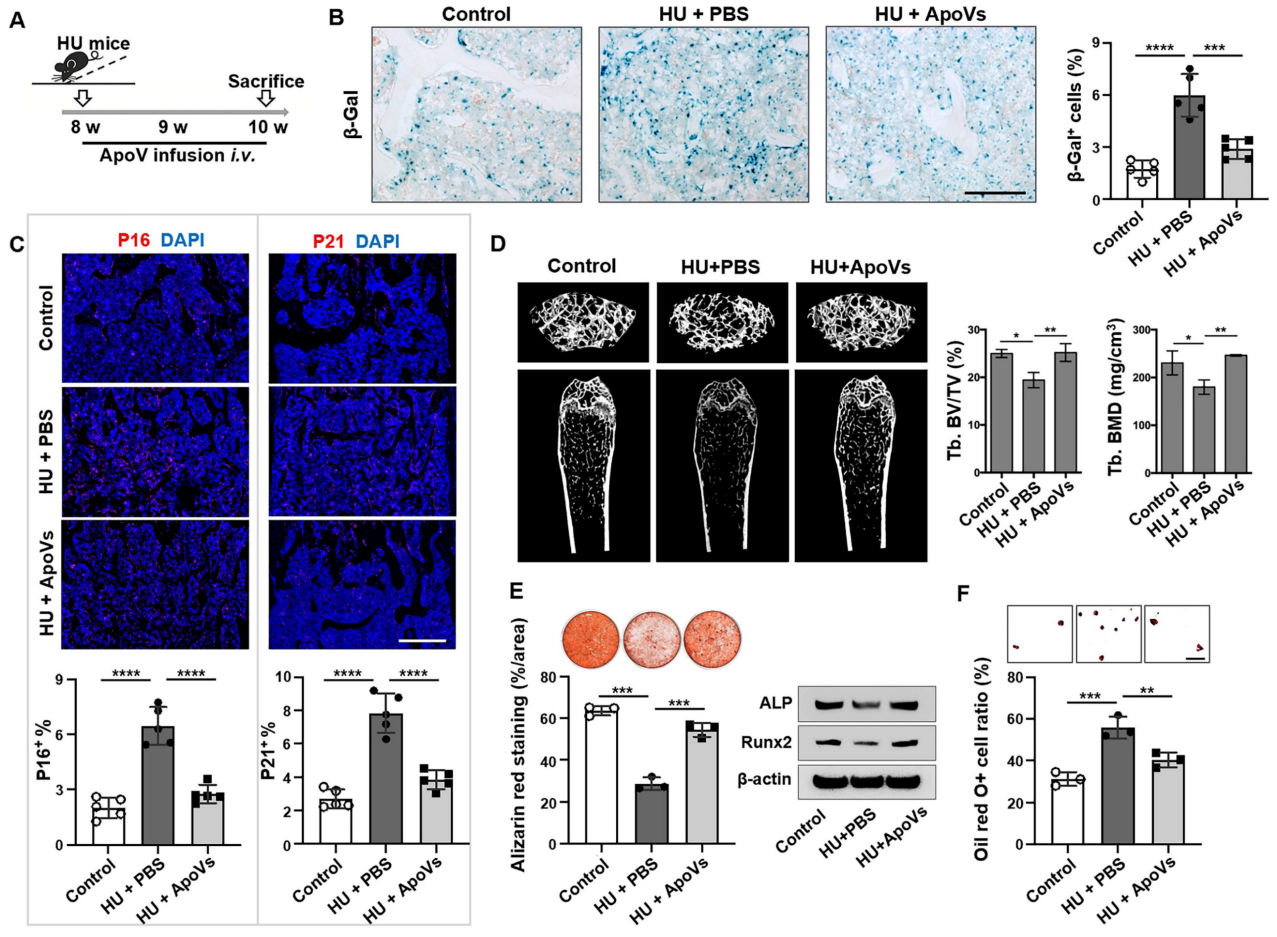
** MSC-apoVs rescue senescent cell accumulation and osteoporosis in mechanical unloading mice.** (**A**) Schematic diagram indicating the study design of exogenous MSC-apoV treatment for hindlimb unloading mice: 8-week-old male C57BL/6 mice were randomized to receive either vehicle or MSC-apoV treatment (twice weekly by systemic infusion) for 2 weeks,* n* = 5. (**B**) SA-β-gal staining of senescent cells in the distal femurs showed that MSC-apoV treatment significantly reduced the number of SA-β-gal^+^ cells in hindlimb unloading mice when compared to vehicle control group. Right panel shows quantification of SA-β-gal^+^ positive cells. Scale bar, 200 μm. (**C**) Immunofluorescence staining showed that MSC-apoV treatment significantly reduced the p16^+^ and p21^+^ cells in the distal femurs of hindlimb unloading mice when compared to the vehicle control group. Lower panel showed quantification of p16 and p21 positive cells, respectively, *n* = 5. Scale bar, 500 μm. (**D**) After 2 weeks of MSC-apoV treatment, the femurs of hindlimb unloading mice showed significantly increased bone mineral density (BMD) and bone volume/total volume (BV/TV), as assessed by microCT. (**E**) After 2 weeks of MSC-apoV infusion, MSCs from hindlimb unloading mice showed significantly increased capacity to form mineralized nodules, as assessed by alizarin red staining. *n* = 3. (**F**) MSCs from apoV-treated hindlimb unloading mice showed significantly decreased capacity to differentiate into adipocytes under adipogenic inductive culture conditions, as assessed by Oil red O staining. *n* = 3. Scale bar, 100 μm. All data are shown as means ± SD. *P < 0.05, **P < 0.01, ***P < 0.001, and ****P < 0.0001.

Since we found that mechanical unloading reduced the production of apoVs in the bone marrow, we attempted to examine whether exogenous apoVs could rescue unloading phenotypes in HU mice. We used STS to induce apoptosis of murine bone marrow MSCs and subsequently isolated apoVs by sequential centrifugation as described previously (Figure S4A)^26^. Under TEM, MSC-derived apoVs appeared as a double-membrane spherical structure (Figure S4B). We confirmed that these apoVs expressed apoV-specific surface marker, as assayed by Western blot (Figure S4C). Nanoparticle tracking analysis showed that 78.86% of MSC-apoVs were 50-250 nm in diameter (average size 164.6 nm), and their membrane potential was -36.17 mV (Figure S4D, E). apoVs (5 × 10^9^ particles) were intravenously infused twice a week for 2 weeks in HU mice (Figure 6A). We found that replenishing apoVs during HU significantly decreased the number of SA-β gal-positive cells in the bone tissue (Figure 6B). Furthermore, we showed that apoV infusion significantly reduced the number of p16^+^, p21^+^, cleaved caspase 3^+^, and cleaved caspase 8^+^ cells in unloading bone tissue compared to vehicle-treated control group (Figure 6C, Figure S5). We further showed that apoV infusion ameliorated the osteopenia phenotype in HU mice, as assessed by micro-CT analysis (Figure 6D). Histological analysis showed that apoV infusion significantly rescued the trabecular bone loss. (Figure S6A). In addition, impaired MSCs from HU mice were also rescued, as demonstrated by increased BrdU labeling and population doubling rates (Figure S6B, C), enhanced mineralized nodule formation, elevated expression of Runx2 and ALP, and decreased adipocyte formation (Figure 6E, F). These data suggest that exogenous apoV infusion offers a therapeutic effect to rescue senescent cell accumulation and osteopenia in HU mice.

### ApoV treatment induces senescent cell apoptosis through restoring calcium influx

**Figure 7:**
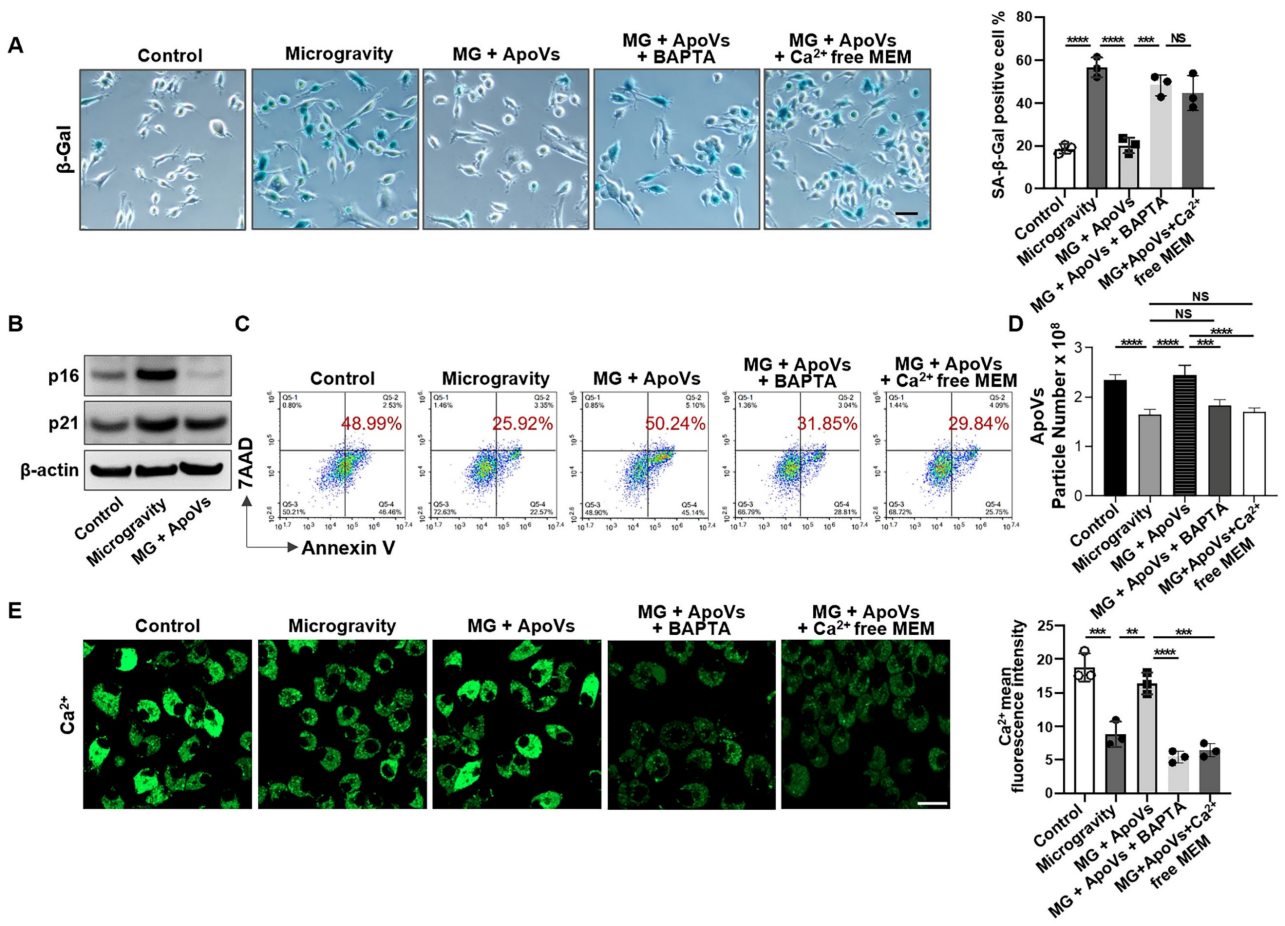
** MSC-apoVs induce senescent cell apoptosis through restoring calcium influx.** (**A**) SA-β-gal staining of senescent cells showed that MSC-apoV treatment rescued the increased number of SA-β-gal^+^ MSCs under microgravity *in vitro*,* n* = 3. Scale bar, 25 μm. (**B**) Western blot analysis showed that MSC-apoV treatment decreased the expression levels of p16 and p21 in MG-MSCs. (**C**) MSC-apoV treatment rescued the decreased apoptosis of starvation-induced MSCs under microgravity. Representative flow cytometry plots and frequency of MSCs under normal gravity, microgravity, and MSC-apoV treatment during starvation in serum-free medium for 48 hours. *n* = 3. (**D**) Nanoparticle tracking analysis showed that exogenous MSC-apoV treatment rescued the reduced particle numbers of apoVs derived from MSCs under microgravity, *n* = 3. (**E**) MSC-apoV treatment significantly rescued the decreased intracellular Ca^2+^ under microgravity. Representative fluorescence images of intracellular Ca^2+^ in MSCs under normal gravity, microgravity, and MSC-apoV treatment during starvation for 48 hours. Cytosolic Ca^2+^ was measured using Fluo-8AM. Scale bar, 20 μm. All data are shown as means ± SD. *P < 0.05, **P < 0.01, ***P < 0.001, and ****P < 0.0001. NS, not significant.

Given that MSC-derived apoVs repressed senescent cell accumulation induced by mechanical unloading *in vivo*, we attempted to further reveal the potential effects of apoVs on MG-MSC populations *in vitro*. After 7 days of recovery phase following 48h of exposure to MG or NG, MSCs were assessed for senescent phenotype using the CCK8 assay, β-gal staining, and western blotting. We found that MSCs under MG exhibited lower bioactivity and a higher number of senescent cells compared to MSCs under NG (Figure S7). When apoptosis was induced by starvation conditions, increased SA-β-gal-positive cells were observed when exposed to MG (Figure 7A). Western blot analysis revealed up-regulated expression of p16 and p21 in MG-MSCs compared to NG-MSCs (Figure 7B). However, we detected a significant decrease in the expression of senescence markers including SA-β-gal, p16 and p21 proteins after co-culturing with apoVs (Figure 7A, B). Importantly, we used flow cytometric analysis to reveal that apoV treatment recovered the number of Annexin V-positive apoptotic cells (Figure 7C). Furthermore, apoV treatment rescued the production of apoVs from MG-MSCs (Figure 7D). These data suggest that apoVs are capable of rescuing the apoptotic resistance of MSCs induced by MG.

Next, we sought to determine how apoVs restore the normal apoptosis of senescent cells under MG. We found that apoV treatment significantly restored intracellular Ca^2+^ in MG-MSCs (Figure 7E). This elevation was absent when the medium was deprived of Ca^2+^ or treatment with the intracellular calcium chelator BAPTA (1 μM, 24-hour) (Figure 7A, C-E), demonstrating that the elevated Ca^2+^ was due to Ca^2+^ influx rather than Ca^2+^ release from storage.

### ApoV treatment rescues impaired calcium influx *via* restoring Piezo1 expression

**Figure 8:**
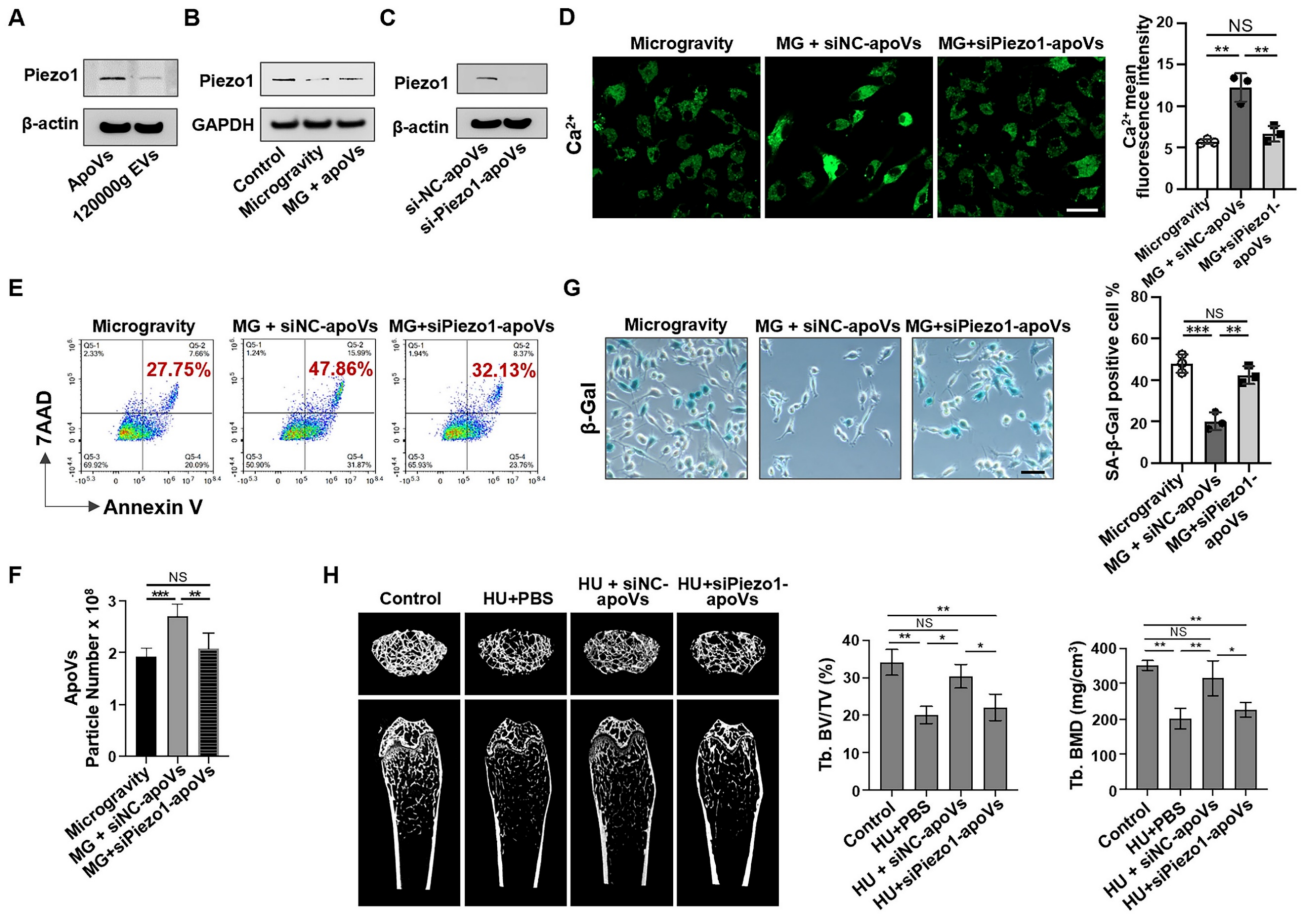
** MSC-apoVs rescue impaired calcium influx *via* restoring Piezo1 expression.** (**A**) Western blot showed the expression of Piezo1 by apoVs. (**B**) When MSC-apoVs were used to treat MSCs under microgravity, upregulation of Piezo1 was observed as assessed by Western blot. GAPDH was used as a protein loading control. (**C**) Western blot showed that the level of Piezo1 in apoVs derived from Piezo1 siRNA-treated MSCs was significantly reduced when compared to the control group. (**D**) Representative fluorescence images of intracellular Ca^2+^ showed that apoVs derived from Piezo1 knockdown MSCs (si-Piezo1-apoVs) failed to rescue decreased intracellular Ca^2+^ in MSCs under microgravity. Scale bar, 20 μm. (**E**) Representative flow cytometry plots showed that si-Piezo1-apoVs failed to rescue the decreased apoptotic rate of MSCs under microgravity when compared to the control group. (**F**) Nanoparticle tracking analysis showed that si-Piezo1-apoVs failed to rescue the reduced number of apoVs from MSCs under microgravity when compared to the control group. (**G**) SA-β-gal staining of senescent cells in MSCs showed that si-Piezo1-apoVs failed to rescue increased SA-β-gal^+^ cells under microgravity compared to the control group. Scale bar, 25 μm. (**H**) Compared to the freely moving WT group, the femurs of HU mice showed significantly reduced bone mineral density (BMD) and bone volume/total volume (BV/TV), as assessed by micro-CT (*n* = 5). After 2 weeks of MSC-apoV treatment, the reduced BMD and BV/TV in the femurs of HU mice were rescued (*n* = 5), but si-Piezo1-apoVs failed to rescue reduced BMD and BV/TV (*n* = 5). All data are shown as means ± SD. *P < 0.05, **P < 0.01, ***P < 0.001, and ****P < 0.0001. NS, not significant.

Since apoV treatment can restore Ca^2+^ influx, we reasoned that certain proteins enriched in apoVs might drive this effect. To explore this, membrane proteins from apoVs and 120000g EVs were extracted to assess Piezo1 expression via Western blotting, which revealed that Piezo1 was significantly enriched in apoVs compared to 120000g EVs (Figure 8A). Considering that mechanical unloading inhibited Piezo1 expression in bone marrow cells, we hypothesized that Piezo1 carried by apoVs may contribute to their therapeutic effects. Notably, in a co-culture system, MSC-derived apoVs rescued the decreased Piezo1 expression in MG-MSCs (Figure 8B). To determine if Piezo1 contributed to apoV-mediated effects, we downregulated Piezo1 expression in mBMMSCs and then isolated si-Piezo1-apoVs to incubate them with MG-MSCs. Western blotting analysis confirmed low Piezo1 expression levels in si-Piezo1-apoVs. (Figure 8C). Notably, compared to the positive control (si-NC-apoVs), the Ca^2+^ influx was markedly suppressed in the group co-cultured with si-Piezo1-apoVs (Figure 8D). In addition, compared to si-NC-apoVs, si-Piezo1-apoVs failed to rescue apoptotic resistance* in vitro*, as evidenced by a lack of significant changes in the apoptotic rate (Figure 8E), the number of apoVs (Figure 8F), and the number of SA-β-gal-positive cells (Figure 8G). After 2 weeks of si-NC-apoVs infusion, the decreased bone volume/tissue volume (BV/TV) and bone mineral density (BMD) in the distal femur of HU mice were restored as assessed by microCT analysis (Figure 8H). However, si-Piezo1-apoVs failed to rescue reduced BMD and BV/TV in HU mice (Figure 8H). These results suggest that Piezo1 carried by apoVs rescues impaired calcium influx and apoptotic resistance induced by mechanical unloading.

## Discussion

All living organisms experience naturally existing mechanical force. Meanwhile, apoptosis and its metabolites, apoptotic vesicles, play pivotal roles in maintaining organ and tissue homeostasis. However, whether mechanical force regulates apoV production is unknown. In this study, we revealed that mechanical unloading induces apoptotic resistance and reduces production of apoVs in bone marrow cells, suggesting that mechanical force is required to maintain apoptotic metabolism. Our data further showed that mechanical unloading leads to the accumulation of senescent cells in the bone marrow, which can be rescued by promoting apoptosis through therapeutic senolysis. These findings suggest that the accumulation of senescent cells caused by decreased apoptotic metabolism is one of the reasons for osteoporosis in unloading mice. Furthermore, we show that systemic infusion of apoVs is capable of eliminating senescent cells in the bone marrow of mechanical unloading mice *via* recovering apoptotic metabolism.

Apoptosis is a highly conserved physiological process that is essential for proper organism development, tissue maintenance, and overall homeostasis. The precise regulation of cell death and clearance is crucial, since both excessive and insufficient apoptotic rates can contribute to the development of various diseases 33-36. Billions of cells undergo apoptosis in the human body daily. Mechanical force is present in all aspects of living systems and has been suspected to play an indispensable role in the body's apoptotic metabolism, but until now, there have been no well-defined mechanisms elucidating how mechanical force regulates the apoptotic process 37. Excessive mechanical force, such as chronic compression of chondrocytes 38 or excessive stretching of pulmonary endothelial cells 39, can trigger apoptosis. Physiologic mechanical stimuli, such as fluid flow-induced shear stress, can protect endothelial cells from apoptosis 40, 41. However, previous studies have found variable responses to mechanical unloading conditions, including increased apoptosis 42-44, decreased apoptosis 45-47 and unchanged apoptosis 48, 49. This may be due to different unloading models, durations of unloading, and methods of detecting apoptosis. In this study, using a hindlimb unloading mouse model and a microgravity cell culture model, we showed that mechanical unloading induces apoptotic resistance in bone marrow cells both *in vivo* and *in vitro*, and reduces the number of apoVs in the circulation. Mechanistically, mechanical unloading results in down-regulated expression of Piezo1 along with reduced calcium influx to further regulate the apoptotic pathway.

In various physiological and pathological situations, cells may undergo apoptosis or enter a death-resistant, non-proliferative and highly secretory state known as senescence. Mechanical stimuli may play a crucial role in determining apoptosis-related responses. We found that mechanical unloading ultimately leads to senescent cell accumulation. This may explain why the bone loss occurs during space travel and long-term bed rest 50-52. Indeed, the most natural treatment for disuse osteoporosis is physical exercise or remobilization (loading) of the affected bones. Unfortunately, most disuse conditions *per se* do not allow remobilization 53. Recently, probiotics have been reported to regulate bone metabolism and treat osteoporosis in animal experiments and clinical trials 54-57. However, the bioavailability and therapeutic effects of probiotics still face enormous challenges due to the limitation of chemical, biological, immune, and mechanical barriers. 58.

Our findings contribute to the expansion of therapeutic applications for senescent cell clearance, a strategy demonstrated to ameliorate various age-associated disease 12-14, 59, 60. For instance, senescent cells have been observed in several bone disorders, such as aging and glucocorticoid-induced disorders 61. Considering that these cells are likely to secrete multiple pathogenic factors, eliminating the source of the SASP could confer benefits beyond neutralizing any single factor, such as VEGF. Therefore, if the senescent cells exacerbate disease common core properties, such as upregulated similar components in antiapoptotic pathways, targeting these pathways with specific senolytic agents, such as dasatinib and quercetin 62, can effectively remove cells that cause tissue dysfunction across numerous age-related diseases occurring as co-morbidities. Therefore, accumulated senescent cells may be new biological targets for elimination in the treatment of mechanical unloading-associated diseases.

EVs can reprogram their target cells through a “Trojan horse” approach, exerting pathogenic or curative effects based on their source cells. Allogeneic EVs obtained from suitable donors are promising as anti-aging treatments and are used for regeneration and rejuvenation in cell-free systems 63, 64. Stem cells-derived EVs carry diverse cargoes that can counteract oxidative stress and inflammation, inducing antiaging effects 65-67. However, the pathologic accumulation of senescent cells is linked to a variety of diseases and age-related conditions across different organ systems. The elimination of senescent cells implicated in disease pathology has the potential not only to arrest ongoing disease progression but also to modify disease by restoring a healthy tissue microenvironment. However, no studies have shown that EVs are able to clear senescent cells. ApoVs are specific apoptotic metabolites that exhibit molecular characteristics quite distinct from those of exosomes and microvesicles 17, 22, 68-70. Our previous work showed that MSC-derived apoVs can induce tumor cell apoptosis and attenuate myeloma bone disease 71. In this study, we found that systemic infusion of MSC-derived exogenous apoVs could eliminate senescent cells *via* restoring Piezo1 expression and calcium influx, and eventually eliminate senescent cells and rescue osteopenia in mechanical unloading mice.

In this study, we found that systemic infusion of MSC-derived exogenous apoVs was able to rescue Piezo1 expression and calcium influx, thereby, rescuing mechanical unloading-induced cellular apoptotic resistance and senescent cell accumulation. This finding provides a potential therapy for a range of diseases caused by a lack of mechanical stimulation. To date, accumulating evidence has documented that MSC-derived apoV transplantation plays an important role in tissue and organ homeostasis maintenance. Our previous study showed that MSCs were able to engulf apoVs via integrin αvβ3 and reuse apoVs-derived ubiquitin ligase RNF146 and miR-328-3p to inhibit Axin1 and thereby activate the Wnt/β-catenin pathway to maintain MSC and bone homeostasis 72. Skin MSCs and hair follicle MSCs are also capable of engulfing exogenous apoVs to nurtures the skin and hair follicle stem cells 73. We also found that apoVs were efferocytosed by macrophages and functionally modulated liver macrophage homeostasis 74. Taken together, the pre-clinical trials conducted thus far have established the recognition of MSC-derived apoV therapeutic effects based on physiological context and developmental origin, representing an indispensable basis for future clinical translational medicine.

Notably, due to the large molecular weight, we failed to overexpress Piezo1 to observe downstream effects. The Piezo1 channel is activated in response to mechanical stimuli generated by movement, mainly through Ca^2+^ regulation of downstream signaling pathways 30, 75. Importantly, Yoda1, a previously identified Piezo1 chemical activator 31, restored the decreased intracellular Ca^2+^ concentration and the reduced apoptotic rate of MSCs under MG conditions. However, we cannot rule out the possibility that other factors, beyond the reduced expression of Piezo1, might be responsible for the observed effects.

In summary, this study reveals a previously unrecognized role of mechanical force in sustaining apoptotic metabolism to eliminate senescent cells. ApoV treatment can effectively rescue apoptotic resistance and eliminate senescent cells.

## Materials and Methods

### Animals

Male C57BL/6J mice were purchased from Sun Yat-sen University in Guangzhou, China. A senescence reporter strain of mice harboring p16^tdTom^ was acquired from Shanghai Model Organisms Center in Shanghai, China. All animal experiments were compliant with the ethics committees of Sun Yat-sen University (SYSU-IACUC-2023-001543). Eight-week-old male mice were subjected to a 2-week continuous hindlimb unloading (HU), following established protocol from a previous study.^76^ In brief, each mouse was housed individually and suspended by the tail using adhesive surgical tape affixed to a chain suspended from a pulley. The mice were positioned at a 30° angle to the floor, with only their forelimbs in contact with the floor, enabling unrestricted movement and free access to food and water. Upon sacrifice, samples of hindlimb bones were collected for the specified analyses.

### Antibodies and reagents

All antibodies, cytokines, kits, and other recourse used in this study are listed in Table S1 (Supporting Information).

### Isolation of mouse MSCs

The isolation and culture of MSCs from the bone marrow of mice were conducted in accordance with our previous study 72. In brief, whole bone marrow cells derived from the femur and tibia were initially seeded, subjected to an overnight incubation, and subsequently washed with phosphate-buffered saline (PBS) to eliminate non-adherent cells. The persisting adherent cells were cultured in alpha-minimum essential medium (α-MEM) supplemented with 20% fetal bovine serum (FBS), 55 μmol·L^-1^ 2-mercaptoethanol, 2 mmol·L^-1^ L-glutamine, 100 U·mL^-1^ penicillin, and 100 μg·mL^-1^ streptomycin under conditions of 37°C in a humidified atmosphere containing 5% CO_2_ and 21% O_2_. MSCs were subjected to digestion with 0.25% trypsin and subsequently passaged for functional experiments following seeding at appropriate cell densities.

### Induction of MSC apoptosis and isolation of apoVs and 120000g EVs

Induction of MSC apoptosis was performed as we reported previously 26. MSCs were washed with 0.1 µm-filtered PBS, and then treated with α-MEM containing 250 nM staurosporine (STS) (Enzo Life Sciences, USA) for 12 hours. ApoVs were isolated from the apoptotic MSCs' medium using sequential centrifugation. In brief, apoptotic cell debris was removed after sequential centrifugation at 800 g for 10 minutes at 4°C and then at 2000 g for 10 minutes at 4°C. The supernatant was then centrifuged at 16,000 g for 30 minutes at 4°C to obtain apoVs, which were subsequently washed with 0.1 μm-filtered PBS.

In addition to the STS induction, MSCs were also induced to apoptosis through starvation, as previously described with modification 26. For starvation-induced apoptosis, MSCs were washed with 0.1 µm-filtered PBS and cultured in a serum-free medium for 48 hours. After treatment, apoVs were isolated using the same process as described above. After apoVs precipitated, the supernatant was centrifuged at 120000 g for 2 hours at 4°C to obtain exosome pellet.

### Isolation of bone marrow extracellular vesicles

The femurs and tibias of mice were dissected, and the bone marrow was then flushed out with 0.1 μm-filtered PBS to obtain single-cell suspension, followed by centrifugation of 1500 g for 5 minutes at 4°C to remove cells. The procedure for isolating bone marrow vesicles from the supernatant is identical to the aforementioned method used for extracting apoVs and 120000g EVs.

### Transmission electron microscopy

Transmission electron microscopy (TEM) assay was conducted according to previous report 71. In brief, apoVs were added in the surface of copper grids and then stained with uranyl acetate treatment for 3 minutes. Subsequently, the images were captured using JEM-1200EX TEM (JEOL, Japan).

### Proteomic analysis

Bone marrow EV samples were extracted from six independent mice. For proteomic analysis, mass spectrometry (MS) data were acquired using Q-Exactive HF instrument (Thermo Fisher Scientific, San Jose, CA) in both data independent acquisition (DIA) mode and data dependent acquisition (DDA) mode. Data quality was assessed through examination of the intra-group coefficient of variation (CV), principal component analysis (PCA), and quantitative correlation of the samples. Proteins were identified through comparison with the UniProt database, employing a false discovery rate (FDR) set at 0.01 for both peptides and proteins during the identification process. Proteins exhibiting significant changes in bone marrow EVs, with a fold change greater than 1.5 and an adjusted *P* value less than 0.05, were selected for further functional analysis utilizing the KEGG and GO databases.

### Microgravity simulation

The Rotating Wall Vessel Bioreactor (RWV) (Synthecon Inc., Texas) is a horizontally oriented rotary cell culture system (RCCS). RCCS is a horizontally rotated cell culture vessel. A silicone membrane is located on the central axis of a rotation chamber to diffuse gases necessary for cell growth without creating turbulence. The solid body rotation of the chamber causes a reduction of the medium's shear stress 77. Mouse bone marrow cells cultured in RCCS with α-minimum essential medium (MEM) free of bubbles was rotated at 16 rpm for 24 or 48 h to simulate a microgravity (0.008 g) environment (MG), in a humidified incubator at 37 °C with 5% CO_2_. Ground based cells cultured under normal gravity (NG) served as control 78.

### Chemical treatments

A cocktail comprising dasatinib (D; 5 mg/kg) (LC Laboratories, USA) and quercetin (Q; 50 mg/kg) (Cayman Chemical Company INC, USA) was dissolved in PBS, and administered via oral gavage on days 0, 7, and 14 post-HU, with vehicle being the control.

### Osteogenic differentiation

To assess the osteogenic differentiation potential, MSCs were cultured in osteogenic inductive medium containing 2 mmol·L^-1^ β-glycerophosphate (Sigma-Aldrich, USA), 10 nmol·L^-1^ dexamethasone (Sigma-Aldrich, USA), and 100 μmol·L^-1^ L-ascorbic acid phosphate (Wako, Japan). Following a 4-week induction, *in vitro* mineralization was evaluated through 1% Alizarin Red S (Sigma-Aldrich, USA) staining. The positively stained regions were quantified as percentages of the total area using ImageJ software (National Institute of Health, USA).

### Adipogenic differentiation

To assess the adipogenic differentiation potential, MSCs were cultured in adipogenic inductive medium containing 10 μg·mL^-1^ insulin (Sigma-Aldrich, USA), 60 μmol·L^-1^ indomethacin (Sigma- Aldrich, USA), 100 nmol·L^-1^ L- ascorbic acid phosphate (Wako, Japan), 500 nmol·L^-1^ isobutylmethylxanthine (Sigma-Aldrich, USA), and 500 nmol·L^-1^ hydrocortisone (Sigma-Aldrich, USA). Following a 7-day induction, lipid droplets were evaluated through Oil Red O (Sigma-Aldrich, USA) staining. The positively stained cells were quantified as percentages of the total cells using ImageJ software.

### Colony-forming unit (CFU) assay

Colony-forming units (CFU) generated by MSCs were assessed according to a previous study.^76^ In brief, 1.5 × 10^6^ all nucleated cells (ANCs) derived from the bone marrow were seeded in 60 mm dishes and cultured for 16 days. Subsequently, the colonies were washed with PBS, fixed using 2% paraformaldehyde, and stained with 0.5% toluidine blue solution (Sigma-Aldrich, USA).

### BrdU cell proliferation assay

MSC proliferation analysis was performed using BrdU labeling. MSCs were seeded onto 8-well chamber slides at a concentration of 2 × 10^4^ cells per well. After adherence, the medium was supplemented with BrdU labeling reagent (Invitrogen, USA) at a dilution of 1:100 for 48 hours. Subsequently, the cells were fixed using 70% ethanol, denatured with 2 N HCl, and stained with a BrdU Staining Kit (Invitrogen, USA). Fluoroshield mounting medium containing DAPI (Abcam, UK) was used for counterstaining and mounting.

### Flow cytometric analysis

The flow cytometry analysis was performed using flow cytometry (ACEA NovoCyte^TM^). For MSC characterization, MSCs were incubated with CD29-PE, CD44-PE, CD90-PE, CD34-PE, and CD45-PE at 4°C for 30 minutes. For apoptosis assessment, cells underwent two washes with Annexin V binding buffer (#422201, BioLegend) and were subsequently incubated with Annexin V-FITC and 7AAD for 15 minutes at room temperature. Data was analyzed by NovoExpress^TM^ software.

### Nanoparticle tracking analysis

ZetaView PMX120 (Particle Metrix, Germany) was used to evaluate concentration of EVs. EVs were resuspended in 1ml PBS, and then diluted with ultrapure water at a ratio of 1:1000-1:10000. Then the samples were injected into the machine and analyzed by ZetaView software 8.02.31.

### Calcium flux assay

Cytosolic Ca^2+^ ([Ca^2+^]i) was measured using Fluo-8AM (#ab142773, Abcam) as the calcium indicator. MSCs were incubated with a buffer solution containing 5 μM Fluo-8 for 30 minutes at 37°C, then washed with Hanks and HEPES buffer (HHBS). Imaging was conducted using Zeiss Elyra 7 with Lattice SIM (Zeiss, German).

### SA-β-galactosidase staining

SA-β-galactosidase (SA-β-gal) is a marker for identifying senescent cells. The cellular senescence assays of frozen sections and MSCs were conducted following the protocol provided with the Cellular Senescence Assay Kit (Merck Millipore). Tissues were incubated in SA-β-Gal staining solution for 12 hours, while cells were incubated 6 hours at 37°C without CO_2_.

### Histological and immunohistochemical staining

Hematoxylin and eosin (H&E) staining was conducted using commercial staining kits (Solarbio, Beijing, China) according to the manufacturer's instructions. For immunohistochemical staining, the bone tissue sections were incubated with primary antibody overnight at 4°C followed by secondary antibody staining. Then, the slides were mounted with mounting medium containing DAPI. The images were captured by a Zeiss LSM 900 confocal microscope and analyzed using ImageJ software.

### MicroCT imaging and analysis

Mouse femurs were collected and examined using a Venus MicroCT (PINGSENG Healthcare, China) with scanning parameters set at a tube voltage of 90 kV, tube current of 70 μA, and voxel size of 13 μm. Avatar software (PINGSENG Healthcare, China) was used for data visualization and analysis. The region of interest is defined as a bone area located 1 mm below the growth plate, extending for a length of 0.5 mm. Bone mineral density (BMD) and the ratio of bone volume to total volume (BV/TV) were calculated for each specimen.

### Western blot

The samples were lysed using a protein extraction kit with protease and phosphatase inhibitors. After quantification with a BCA kit, 20 μg of protein of each sample was loaded onto SDS-polyacrylamide electrophoresis gels and subsequently transferred to PVDF membranes (Millipore). The membranes were blocked with 5% BSA in TBST for 1 hour and then incubated at 4°C overnight with Caspase-3, cleaved Caspase-3, Caspase-8, cleaved Caspase-8, Fas, Lamin B1, Syntenin-4, Calreticulin, Piezo1, p16, p21, Bcl-xl, ALP, Runx2, β-actin, or GAPDH primary antibodies. After incubation with HRP-conjugated secondary antibodies for 1 h, the protein bands were visualized using SuperSignal West Pico Chemiluminescent Substrate (Thermo Fisher) and evaluated with a gel imaging system (Bio-Rad, USA).

### Statistics

All data were expressed as mean ± standard deviations (SD). Comparisons between two groups were analyzed using independent unpaired two-tailed Student's *t*-tests, and comparisons between more than two groups were analyzed using two-way ANOVA with Dunnett's test, or one-way ANOVA with Tukey's test. *P* values less than 0.05 were considered statistically significant.

## Supplementary Material

Supplementary figures and table.
