# Supplementary material for: Apoptotic vesicle-mediated senolytics requires mechanical loading
Source: Theranostics. 2024 Aug 6;14(12):4730–46. doi: 10.7150/thno.98763 (PMC11373628; doi:10.7150/thno.98763)
Supplement: Supplementary file 1 — Supplementary figures and table. [file thnov14p4730s1.pdf]

Supporting Information

Apoptotic vesicle-mediated senolytics requires mechanical loading

Zhulin Xue[1,2], Yexiang Jiang[2], Bowen Meng[2], Lu Lu[2], Meng Hao[2], Yi Zhang[1], Songtao Shi[2,3], Zili Li [1,*], Xueli Mao [2,3,*]

[1]Department of Oral and Maxillofacial Surgery, Peking University School and Hospital of Stomatology & National Center for Stomatology & National Clinical Research Center for Oral Diseases & National Engineering Research Center of Oral Biomaterials and Digital Medical Devices, Beijing 100081, China

[2]Hospital of Stomatology, Guanghua School of Stomatology, Sun Yat-sen University, South China Center of Craniofacial Stem Cell Research, Guangdong Provincial Key Laboratory of Stomatology, Guangzhou 510055, China

[3]Key Laboratory of Stem Cells and Tissue Engineering (Sun Yat-Sen University), Ministry of Education, Guangzhou 510080, China

[4]These authors contributed equally

[*] **Corresponding authors:**

Prof. Xueli Mao, South China Center of Craniofacial Stem Cell Research, Guanghua School and Hospital of Stomatology, Sun Yat-sen University, 74 Zhongshan 2Rd, Guangzhou, Guangdong, 510080, China. Tel: 86-020-83812602. E-mail: maoxuel@mail.sysu.edu.cn.

Prof. Zili Li, Department of Oral and Maxillofacial Surgery, Peking University School and Hospital of Stomatology & National Center for Stomatology & National Clinical Research Center for Oral Diseases & National Engineering Research Center of Oral Biomaterials and Digital Medical Devices, No.22, Zhongguancun South Avenue, Haidian District, Beijing, 100081, China. Tel: 86-010-82195296. E-mail: kqlzl@sina.com.

Supplementary Figures

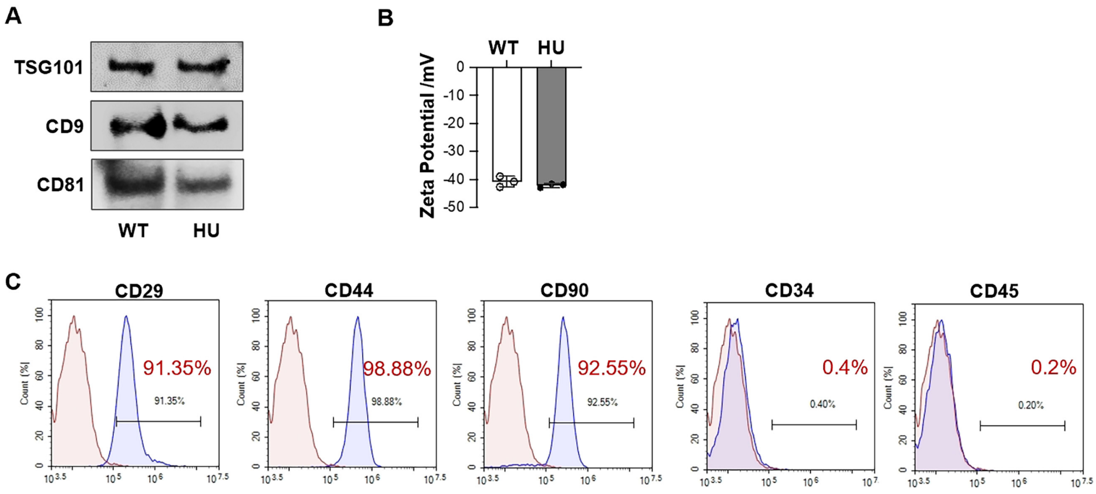

Figure S1. Characteristics of bone marrow EVs. (**A**) Western blot analysis showing the EV markers in bone marrow EVs. (**B**) Nanoparticle tracking analysis showing the membrane potential of bone marrow EVs. (**C**) Flow cytometric analysis showed the markers of MSCs (CD29, CD44, and CD90), hematopoietic markers (CD34 and CD45).

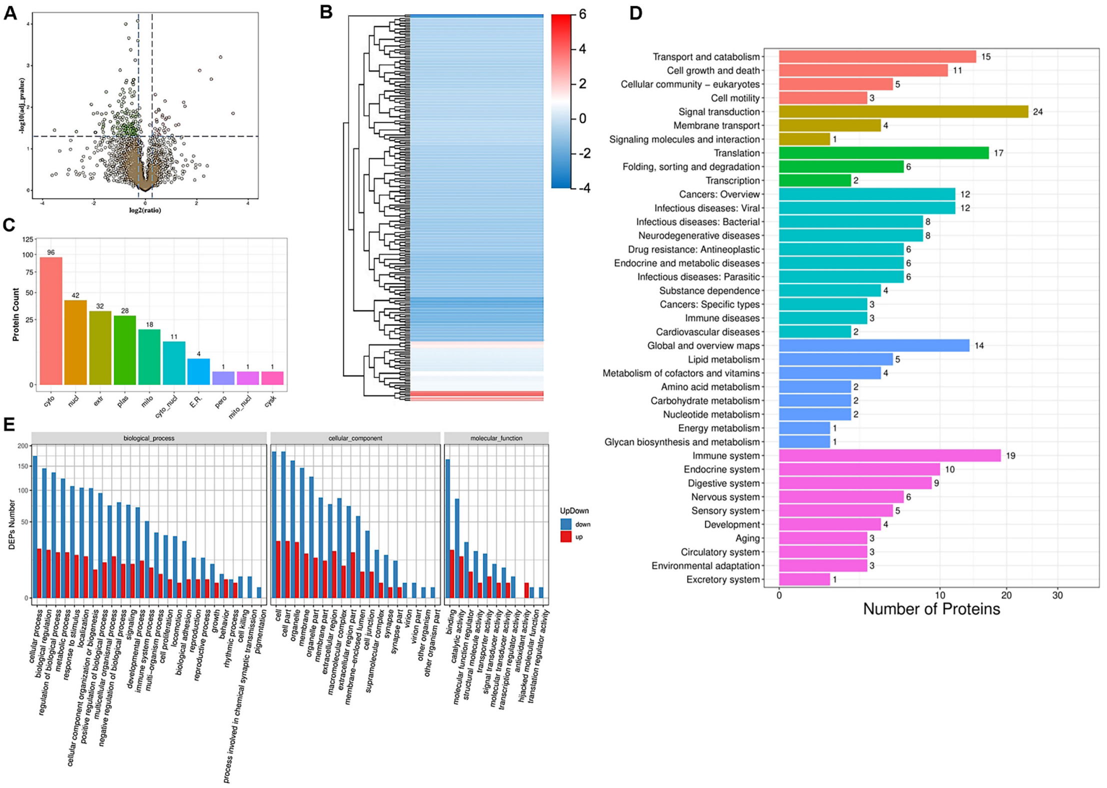

Figure S2. Mechanical unloading alters protein content in bone marrow EVs. (**A**) Volcano plots showing significantly upregulated (red dots) and downregulated (green dots) protein expression in HU-EVs compared to WT-EVs. Fold change >= 1.2 and adjusted *P* value < 0.05 were used to obtain differentially expressed proteins (DEPs). (**B**) Clustering heatmap of DEPs in HU-EVs was different from WT-EVs. (**C**) Subcellular localizations of DEPs in HU-EVs compared to WT-EVs. Cyto, cytosol; Nucl, nucleus; Extr, extracellular; Plas, plasma membrane; Mito, mitochondria; Cyto_nucl, cytosol-nucleus; E.R., endoplasmic reticulum; Pero, peroxisome; Mito_nucl, mitochondria-nucleus; Cysk, cytoskeleton. (**D**) Differential protein pathway classification of HU-EVs compared to WT-EVs. (**E**) Gene ontology (GO) analysis of DEPs in HU-EVs compared to WT-EVs, categorized into “Biological process”, “Cellular component”, and “Molecular function”. HU-EVs, the bone marrow EVs from hindlimb unloading mice; WT-EVs, the bone marrow EVs from freely moving wildtype mice.

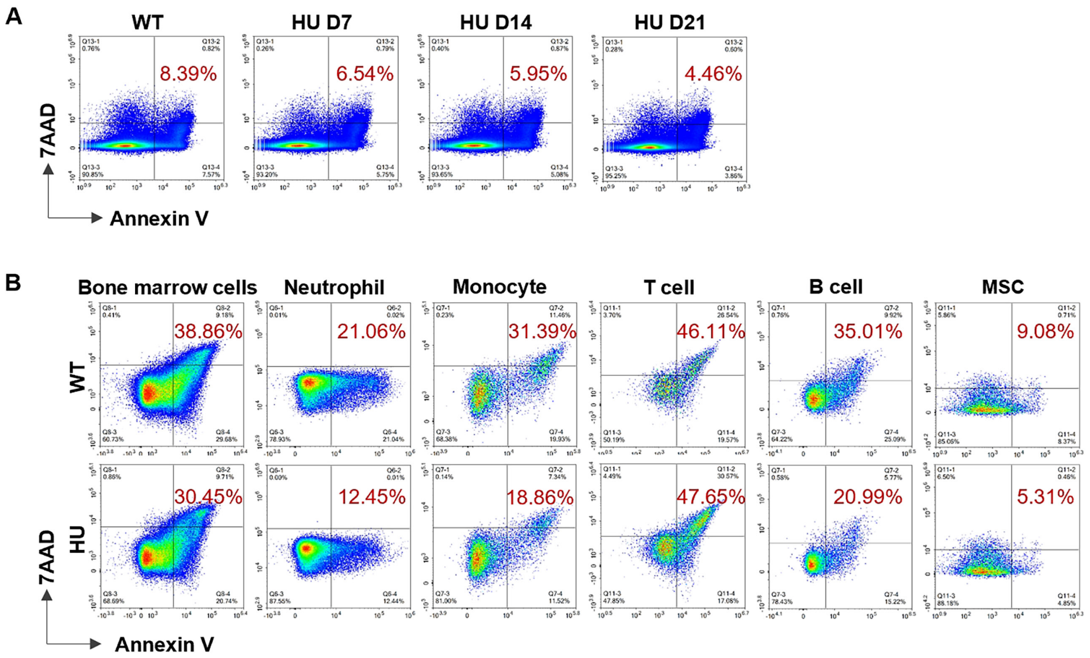

Figure S3. Mechanical unloading induces apoptotic resistance of bone marrow cells. (**A**) Hindlimb unloading repressed bone marrow cell apoptosis in a time-dependent manner. Representative flow cytometry plots and frequency of total bone marrow cells after 0, 7, 14, and 21 days of hindlimb unloading. *n* = 3. (**B**) Hindlimb unloading repressed apoptosis in subsets of bone marrow cells. Representative flow cytometry plots and frequency of $CD11b^+Ly6G^+$ neutrophils, $CD11b^+Ly6G^-Ly6C^+$ monocytes, $CD3^+$ T cells, $B220^+$ B cells, and $Sca1^+$ MSCs.

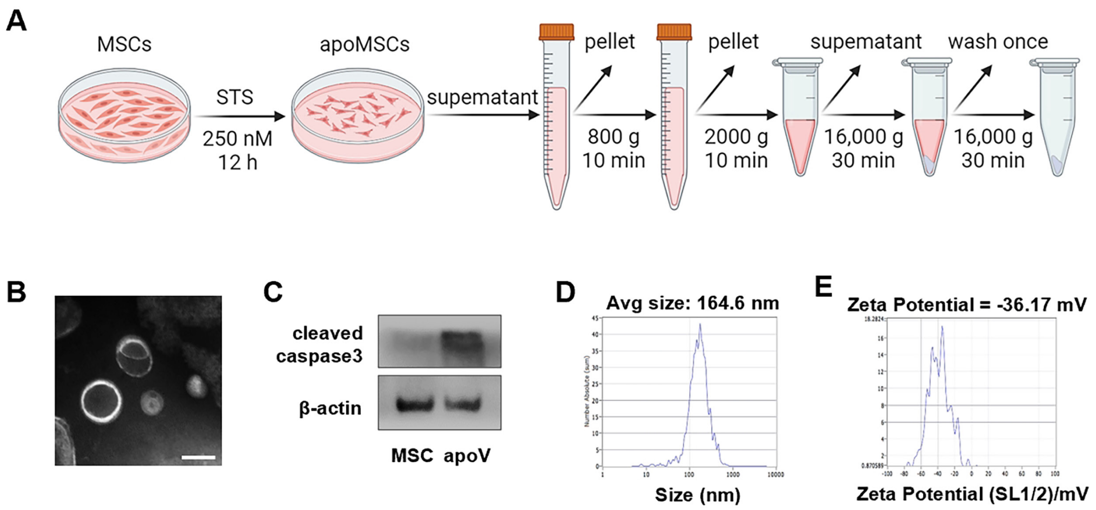

Figure S4. Characteristics of apoptotic vesicles (apoVs) derived from MSCs. (**A**) Scheme illustrating the isolation procedure of apoVs from MSCs. (**B**) Transmission electron microscopy analysis of mouse MSC- derived apoVs. Scale bar, 100 nm. (**C**) Western blot analysis showed MSC-apoVs were positive for cleaved Caspase-3. (**D** and **E**) Nanoparticle tracking analysis of apoVs by Zetaview showed a mean diameter of 164.6 nm and -36.17 mV membrane potential of these vesicles. The Zetaview was calibrated using standard beads of 100 nm. PBS was used as a negative control.

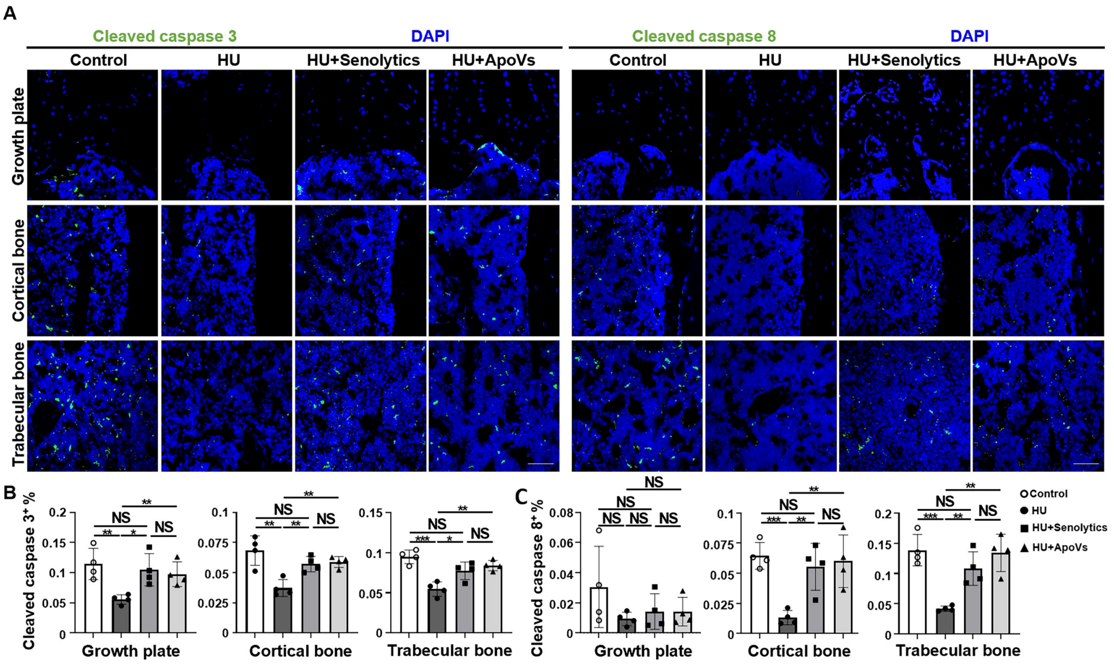

Figure. S5. Senolytics and MSC-apoVs rescue apoptosis resistance in unloading mice. (**A**) Representative immunostaining showed that hindlimb unloading resulted in a reduced apoptotic rate in the bone marrow compared with that in wildtype mice and unloading mice treated with Senolytics and ApoVs. Scale bars, 50 μm. (**B** and **C**) Quantification of cleaved caspase 3 and cleaved caspase 8 positive cells, respectively, *n* = 4.

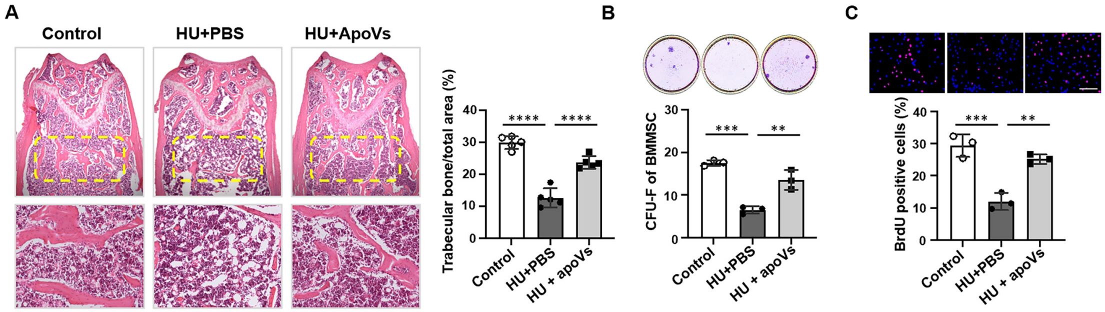

Figure S6. MSC-apoVs rescue osteoporosis and MSC deficiency in mechanical unloading mice. (**A**) Representative H&E staining showed that hindlimb unloading resulted in a reduced trabecular bone area (yellow circled area) compared to freely moving wildtype mice. MSC-apoV treatment significantly increased the trabecular bone area. Scale bars: 1 mm for upper panel and 100 μm for lower panel. (**B** and **C**) After 2 weeks of MSC-apoV infusion, BrdU labeling and colony formation assays showed that the decreased proliferation and population doubling rates were rescued in hindlimb unloading MSCs. $n = 3$. Scale bar, 100 μm.

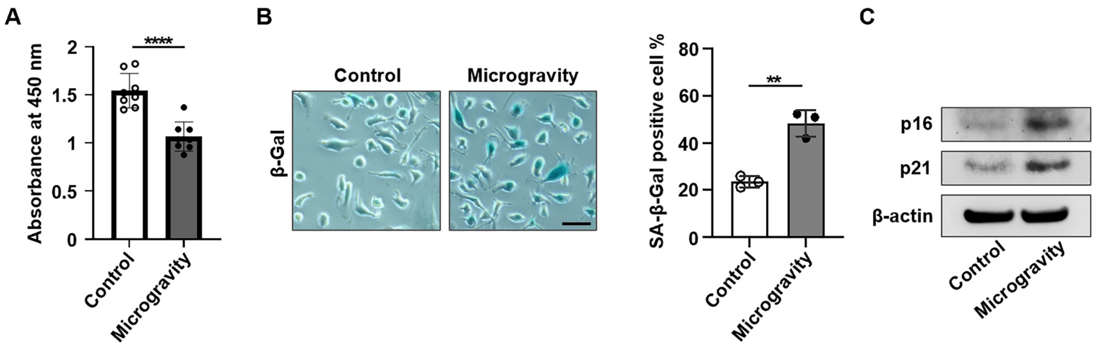

Figure. S7. Microgravity contributes to senescent cell phenotype.

(A) CCK8 assay showed that microgravity resulted in a decreased bioactivity compared with that in control group. **(B)** SA-β-gal staining of senescent cells increased in MG compared to control after 7 days of recovery phase following 24 h of exposure to MG or normal gravity. **(C)** Western blot analysis showed that MG upregulated p16 and p21 expression in MSCs. β-actin was used as a protein loading control.

Supplementary Table

Table S1. Antibodies and Reagents

**Reagent or resource**	**Source**	**Identifier**
**Antibodies**		
Anti-Cleaved Caspase-3 -Rabbit	CST	9664s
Anti-Caspase-3 -Rabbit	CST	9662s
Anti-Cleaved Caspase-8 -Rabbit	CST	8592s
Anti-Caspase-8- Rabbit	Abcam	ab25901
Anti-Calreticulin -Rabbit	CST	12238s
Anti-β actin-Mouse	Sigma	A5441
Anti-P21-rabbit	Abcam	ab188224
Anti-CDKN2A/p16INK4a-Rabbit	Abcam	ab211542
Anti-Rad5 c-Mouse	Santa Cruz	sc-46692
Anti-Syntaxin-4-Mouse	Santa Cruz	sc-5275
Anti-Lamin B1 -Mouse	Santa Cruz	sc-37000
Anti-Fas-Mouse	Santa Cruz	sc-74540
Anti-Piezo1- Rabbit	Proteintech	15939
Anti-GAPDH-Mouse	Servicebio	GB12002
Anti-ALP- Rabbit	Abcam	ab108337
Anti-RUNX 2- Rabbit	Affinity	AF5186
Anti-TSG101- Rabbit	Abcam	ab125011
Anti-CD9- Mouse	Santa Cruz	sc-13118
Anti-CD81- Mouse	Santa Cruz	sc-70803
FITC Annexin V	Biolegend	640906
FITC anti-mouse B220	Biolegend	103206
FITC anti-mouse Ly6G/Ly6C	Biolegend	108405
PE/Cyanine7 anti-mouse Ly-6A/E (Sca-1)	Biolegend	108114
PE anti-mouse Ly6G	Biolegend	127608
PE anti-mouse CD95 (Fas)	Biolegend	152607
APC-Cy7 anti-mouse CD11b	Biolegend	101226
**Cells and Reagents**	**Source**	**Identifier**
Human bone marrow mesenchymal stem cells (hBMMSCs)	ScienCell	7500

Mounting Medium with DAPI	Abcam	ab104139
SA-β-galactosidase detection Kit	Solarbio	G1580
BrdU Staining Kit	eBioscience	8800-6599-45
Fluo-8AM	Abcam	ab142773
protein extraction kit	Thermo Fisher	78501/ 89842
CellMask™ Deep Red plasma membrane stain	Thermo Fisher	C10046
Dexamethasone	Sigma-Aldrich	D4902
Staurosporine	Enzo Life Sciences	ALX-380-014
TritonTM X-100	Sigma-Aldrich	X100-100ML
α-MEM	Invitrogen	12571-048
Fetal bovine serum (FBS)	Gibco	10270-106
L -glutamine	Invitrogen	35050-061
2-mercaptoethanol	Invitrogen	21985-023
Penicillin-Streptomycin	Invitrogen	15140-122
TrypLETM Express Enzyme	Invitrogen	12605-010
10X Annexin V Binding Buffer	BD Pharmingen	556454
NuPAGE MES SDS Running Buffer	Invitrogen	NP0002
NuPAGE Transfer Buffer	Invitrogen	NP00061
PageRuler™ Prestained Protein Ladder	Invitrogen	26616
Novex™ Sharp Pre-stained Protein Standard	Invitrogen	LC5800
NuPAGE™ LDS Sample Buffer	Invitrogen	NP0007
Lipofectamine^{TM} RNAiMAX Transfection Reagent	Invitrogen	13778
Opti-MEM Medium	Invitrogen	31985070
